# Repetitive Trans-spinal Magnetic Stimulation Suppresses Microglia to Engulf Synapse and Promotes Nerve Repairment via cGAS-STING Signaling Pathway after Spinal Cord Injury

**DOI:** 10.7150/ijbs.114428

**Published:** 2025-09-29

**Authors:** Mudan Huang, Jiawei Di, Na Li, Longyou Xiao, Zhenming Tian, Tianwei He, Mao Pang, Bin Liu, Lei He, Limin Rong

**Affiliations:** 1 Department of Spine Surgery, The Third Affiliated Hospital, Sun Yat-sen University, No. 600 Tianhe Road, Guangzhou, 510630, Guangdong, China.; 2 Guangdong Provincial Center for Quality Control of Minimally Invasive Spine Surgery, No. 600 Tianhe Road, Guangzhou, 510630, China.; 3 Guangdong Provincial Center for Engineering and Technology Research of Minimally Invasive Spine Surgery, No. 600 Tianhe Road, Guangzhou, 510630, China.; 4 Department of Rehabilitation Medicine, The Third Affiliated Hospital, Sun Yat-sen University, No. 600 Tianhe Road, Guangzhou, 510630, Guangdong, China.

**Keywords:** Spinal cord injury, Magnetic stimulation, Microglia, Synapse phagocytosis, cGAS, STING

## Abstract

**Background:** Spinal cord injury (SCI) is a neurological disorder characterized by progressive neuronal death. Notably, microglia-mediated synapse phagocytosis contributes to the disruption of the surviving neuronal network. Recovery of neurological function after SCI largely relies on the activation and remodeling of neural circuits. Magnetic stimulation has been shown to improve the reconstruction of neural synapses and neural circuits. However, the specific mechanisms by which repetitive trans-spinal magnetic stimulation (rTSMS) modulates microglial phagocytosis of synapses in SCI remain unclear.

**Methods:** A modified version of Allen's method was used to establish an SCI model. Structural recovery was assessed using Hematoxylin-eosin and Nissl staining. Neurological function was evaluated through several assessments: the Basso, Beattie, and Bresnahan scale, the modified Rivlin inclined plate test, the horizontal ladder test, thermal pain assessment, motor evoked potential measurements, and gait analyses. Single-cell RNA sequencing was utilized to elucidate the cellular and molecular mechanisms by which rTSMS promotes recovery after SCI. Additionally, western blotting and immunofluorescence staining were performed to measure microglial phagocytosis of synapses and to investigate the expression of components of the cyclic GMP-AMP synthase (cGAS)-STING pathway. Furthermore, the STING agonist 2,3 cGAMP was used to further explore the role of the cGAS-STING pathway in the effects of rTSMS.

**Results:** rTSMS significantly reduced the lesion area and improved functional recovery in rats subjected to SCI, and these changes correlated with enhanced synapse reservation and axon regeneration. Single-cell RNA sequencing identified microglia as the primary target cells that actively respond to rTSMS. Importantly, rTSMS effectively inhibited the phagocytosis of synapses by overactivated microglia via suppressing the cGAS-STING pathway. Moreover, 2,3 cGAMP counteracted the effects of magnetic stimulation on microglia both *in vivo* and *in vitro*.

**Conclusion:** rTSMS mitigates SCI-induced synapse loss and neurological deficits by modulating microglial phagocytosis, a process dependent on the cGAS-STING pathway. These findings provide new insights into the mechanisms by which rTSMS exerts neuroprotective effects in the context of SCI.

## Introduction

Spinal cord injury (SCI) is one of the most devastating traumatic conditions and causes major motor, sensory and autonomic dysfunction 1, 2. Following SCI, primary injury occurs due to physical forces such as compression, shearing, and laceration. More importantly, this is followed by a cascade of secondary injury events that lead to the death of numerous neurons and the loss of synapses, including inflammation, glutamate excitotoxicity, myelin debris accumulation, and glial scarring 3, 4.

Microglia are dynamic macrophages in the central nervous system (CNS) that respond to a wide variety of pathological insults after SCI. Microglia are rapidly activated within three days after SCI (the acute phase). A second wave of microglial activation occurs approximately 14 days post-injury (sub-acute phase) 5. Activated microglia differentiate into two main phenotypes: M1 and M2. M1 microglia exacerbate neuronal injury and hinder cellular repair by upregulating pro-inflammatory cytokines such as tumor necrosis factor α (TNFα), inducible nitric oxide synthase (iNOS), and interleukin 1β (IL-1β) 6. In contrast, microglia of the M2 phenotype mediate neuroprotection and facilitate functional recovery through the release of anti-inflammatory cytokines, including IL-4, IL-10, and arginase 1 (Arg1) 7. In addition to the neuroinflammatory effects induced by activated microglia, Tillmon *et al.* suggested that microglial activation leads to disease-specific patterns of synapse loss in many brain diseases 8. A recent study also indicated that M1 microglia are involved in synapse elimination in rats with depression-like behavior induced by chronic mild stress 9. Moreover, inhibiting microglial phagocytosis in models of ischemic stroke has been suggested to promote the recovery of motor function and reduce brain damage 10. These findings indicate that modulating microglial activation and phagocytic activity may represent a promising therapeutic strategy for SCI.

Repetitive transcranial magnetic stimulation (rTMS), a neuromodulation technique that is used to modulate neuronal electrical potential, has emerged as an effective and noninvasive treatment for SCI in the clinic 11. Numerous studies have confirmed that rTMS alleviates neuron impairment and contributes to the recovery of motor and sensory function in SCI models 12, 13. Notably, the effect of magnetic stimulation on SCI appears to be variable, a phenomenon that is closely related to various parameters (such as intensity, frequency, duration and position) 14. rTMS can partially improve corticomotor connectivity following SCI but fails to form cellular substrates for axon elongation through the lesion site 15, 16. In recent years, repetitive trans-spinal magnetic stimulation (rTSMS), a focal stimulation method derived from rTMS, has been shown to improve the inhibitory environment of axonal regeneration and promote functional recovery after SCI 3. However, the therapeutic effects of rTSMS at different frequencies vary, and few studies have compared the effects of low-frequency and high-frequency rTSMS on SCI. Additionally, the precise cellular and molecular mechanisms underlying these effects remain largely unexplored. Thus, in the current study, we propose the use of single-cell RNA sequencing to screen for potential targets of rTSMS for the treatment of SCI.

In the present study, we demonstrated that low-frequency rTSMS has a greater capacity to alleviate structural disruption and improve neurological function following SCI. Additionally, we found that low-frequency rTSMS promotes synapse reservation and axonal regeneration during the chronic phase. Single-cell RNA sequencing analysis indicated that microglial activation plays a crucial role in the neuroprotective effects of rTSMS. Our results further revealed that rTSMS significantly inhibits SCI-induced microglial activation and alleviates microglia-mediated synapse phagocytosis via the cGAS-STING signaling pathway. These findings may provide new insights into the role of rTSMS in SCI rehabilitation.

## Materials and Methods

### Experimental animals and SCI model

Adult female Sprague-Dawley (SD) rats (specific-pathogen-free, aged 6-7 weeks, weighing 200-230 g) were obtained from the Silaike Laboratory Animal Center in Hunan, China. The rats were housed in the Experimental Animal Center of South China Agricultural University, Guangzhou, China (approval no. SYXK(Yue)2022-0136). All the animals were acclimated in groups of three per cage for seven days prior to surgery and were kept in a standard experimental animal room maintained at 20-26°C, with a 12-hour light/dark cycle and 40-70% relative humidity. The SD rats had free access to food and water. All surgical procedures and animal care protocols were conducted in accordance with the National Institutes of Health (NIH) Guidelines for the Care and Use of Laboratory Animals and were approved by the Experimental Animal Center of South China Agricultural University (approval no. 2022D128).

**Figure 1:**
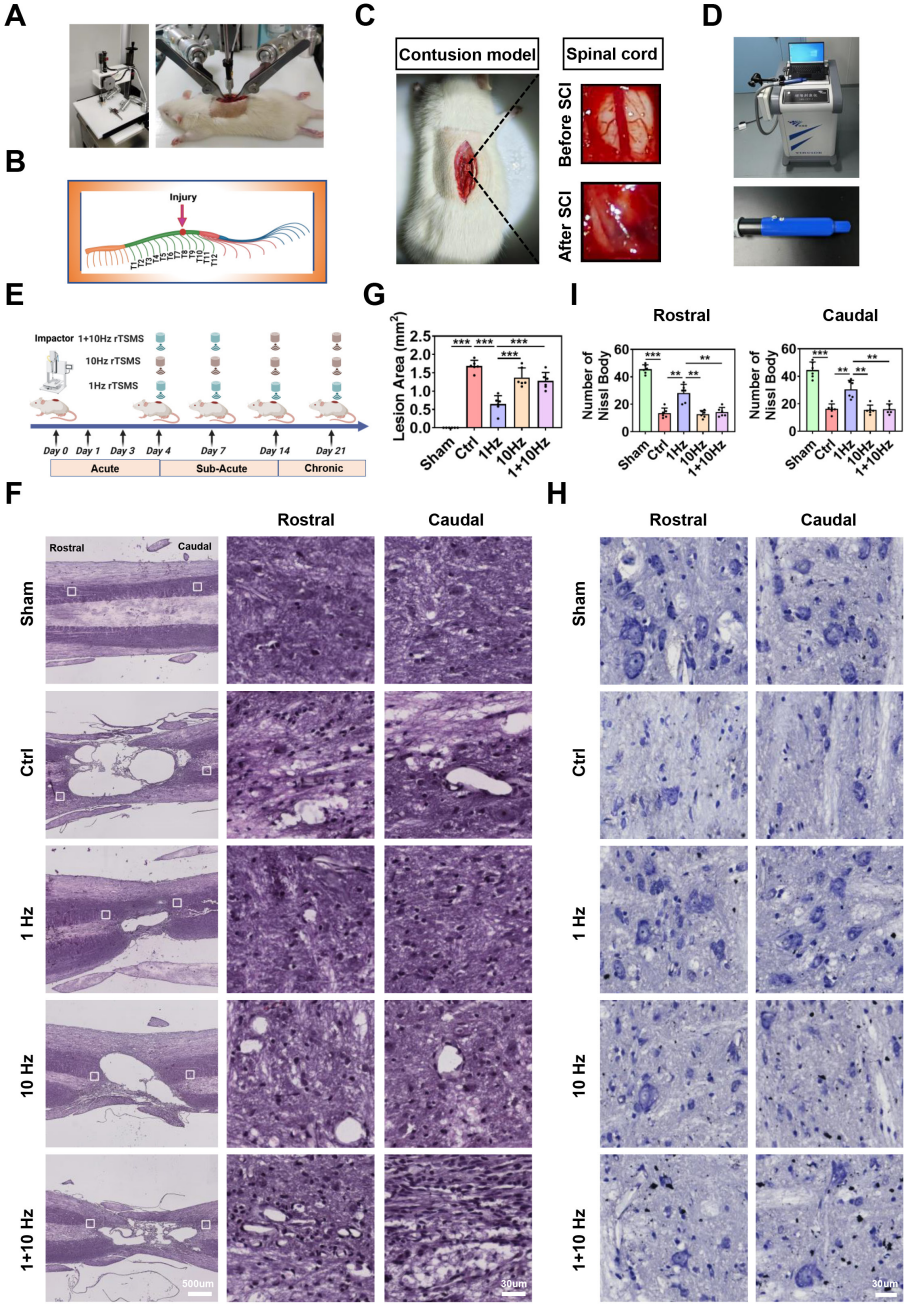
** Low-frequency rTSMS ameliorates lesion area and promotes neuronal survival in rats with SCI.** (A) An SCI animal model was established using an Infinite Horizon Spinal Cord Impactor. (B) A schematic illustration showing the T10 segment. The image was generated with BioRender.com. (C) Representative images of the spinal cord before and after SCI. (D) Images of the rTSMS equipment and the animal special coil. (E) The experimental design was created with BioRender.com. (F-G) Representative images (HE staining) and quantification of the lesion area in rats at 21 days post-SCI, n = 6. (H-I) Representative images (Nissl staining) and quantification of Nissl body at 21 days post-SCI, n = 6. **P < 0.01, ***P < 0.001.

To establish a contusive SCI model, a modified version of Allen's method was used, as described in a previous study 17. Briefly, the animals were anesthetized via intraperitoneal injection of 1% (w/v) pentobarbital sodium (50 mg/kg, Sigma-Aldrich). After hair removal, a dorsal laminectomy was performed to expose the spinal cord. As shown in Fig. 1A-C, the T10 spinal cord segment was impacted using an Infinite Horizon impactor with a force of 200 kilodynes (Precision Systems and Instrumentation, LLC, Versailles, KY, USA). Post-operative care included seven days of antibiotic treatment with ampicillin sodium (60 mg/kg), promoting urination and defecation through abdominal massages (twice daily), and training the hind limbs to prevent pressure sores (twice daily). Animals in the Sham group underwent T10 laminectomy without contusion.

### Animal grouping

The acute phase of SCI in animals was defined as the first three days post-injury. The sub-acute phase encompassed the period from four to 14 days after SCI, whereas the chronic phase referred to the period beyond 14 days 18. To determine the optimal frequency of rTSMS initiated in the sub-acute stage, the experimental animals were randomly assigned to five groups, each consisting of 13 rats: (1) the rats in the Sham group underwent sham surgery (Sham group); (2) the rats in the control group underwent SCI surgery followed by sham magnetic stimulation treatment (Ctrl group); (3) the rats in the 1 Hz group underwent SCI surgery followed by low-frequency rTSMS treatment at 1 Hz initiated in the sub-acute phase (1 Hz group); (4) the rats in the 10 Hz group underwent SCI surgery followed by high-frequency rTSMS treatment at 10 Hz initiated in the sub-acute phase after SCI (10 Hz group); and (5) the rats in the 1+10 Hz group underwent SCI surgery followed by low-frequency rTSMS treatment at 1 Hz (sub-acute stage, days 4-14), followed by high-frequency rTSMS treatment at 10 Hz (chronic stage, days 15-21) (1+10 Hz group).

To evaluate the role of STING in the neuroprotective effects of rTSMS, 2',3'-cyclic guanosine monophosphate-adenosine monophosphate sodium (2,3 cGAMP, a STING agonist; Catalog no. L2880-25MG, Sigma-Aldrich, USA) or vehicle (phosphate-buffered saline, PBS) was administered intranasally one hour prior to each rTSMS treatment. The dose of 2,3 cGAMP used was 500 μg/kg, based on previous studies 19, 20. In this animal experiment, a total of four groups were randomly assigned, with each group consisting of 13 rats: (1) the rats in the Sham group underwent sham surgery (Sham group); (2) the rats in the control group underwent SCI surgery followed by sham magnetic stimulation treatment (Ctrl group); (3) the rats in the rTSMS group underwent SCI surgery and were then treated with PBS and 1 Hz rTSMS (rTSMS group); and (4) the rats in the 2,3 cGAMP group underwent SCI surgery and were then treated with 2,3 cGAMP and 1 Hz rTSMS (2,3 cGAMP group).

### rTSMS treatment

As shown in Fig. 1D, a customized stimulator (YRDCCI, Wuhan, China) was employed to administer rTSMS treatment in this study. The round prototype coil has a diameter of 23 mm, generates peak magnetic fields of 3.5 T, and induces an electric field of 140 V/m. Briefly, the rats were placed in a breathable rodent restraint bag (Braintree Scientific, USA, MA), and the coil was positioned over the spinal cord. rTSMS treatment was initiated on the fourth day and continued until the 21^st^ post-operative day (a total of 18 days). The treatment sessions were conducted from 8:00 a.m. to 12:00 a.m.

To compare the effects of low-frequency and high-frequency rTSMS on SCI, 1 Hz low-frequency rTSMS and 10 Hz high-frequency rTSMS were employed, consistent with a previous report 21. Moreover, Ahmed *et al.* suggested that low-frequency rTSMS should be used in the early phase, whereas high-frequency rTSMS should be applied in the later phase following SCI 22, 23. Therefore, in our experimental groups, one group received low-frequency rTSMS treatment during the sub-acute and chronic phases, another group received high-frequency rTSMS treatment during the sub-acute and chronic phases, and the last group received low-frequency rTSMS treatment during the sub-acute phase and high-frequency rTSMS treatment during the chronic phase. In accordance with our previous study 24, the rTSMS parameters established in this study were as follows: (1) 1 Hz low-frequency rTSMS: 20 pulses per train, 5-second intervals, totaling 30 trains (600 pulses), at 30% maximum output intensity of the machine; (2) 10 Hz high-frequency rTSMS: 20 pulses per train, 5-second intervals, totaling 30 trains (600 pulses), at 30% maximum output intensity of the machine; (3) 1+10 Hz rTSMS: animals in the 1+10 Hz rTSMS group underwent 1 Hz rTSMS during the sub-acute phase (days 4-14), followed by 10 Hz rTSMS during the chronic phase (days 15-21); and (4) sham magnetic stimulation: rats in the control (Ctrl) group experienced the same experimental manipulations and auditory stimuli by using a 90° coil tilt 25. The detailed parameters used for rTSMS and the positioning of the animal coil during the rTSMS and sham magnetic stimulation treatments were shown in Figure S1A-B.

### Hematoxylin-eosin (HE) staining

On the 21^st^ post-operative day, the rats were anesthetized, and cardiac perfusion was performed using 0.9% normal saline (catalog no. P001228, Newprobe, China) followed by 4% paraformaldehyde (PFA, catalog no. UI0127B, UBIO, China). The T9-T11 spinal cord and brain tissues were rapidly collected and fixed in 4% PFA overnight. The tissues were subsequently dehydrated in graded sucrose solutions (10%, 20%, and 30% sucrose). Finally, the tissues were embedded in O.C.T. compound (catalog no. BL557A, Biosharp, China) and sectioned longitudinally into slices using a low-temperature thermostatic microtome (Leica, Germany).

Frozen sections of the spinal cord were stained with HE staining reagent (catalog no. G1120-3, Solarbio, China). According to the manufacturer's instructions, the spinal cord sections were immersed in hematoxylin solution for 5 minutes and then rinsed with ultrapure water for 10 minutes before being immersed in eosin solution for another 5 minutes. The sections were subsequently dehydrated using graded ethanol solutions (80%, 90%, 95%, and 100%) and cleared with xylene for 30 minutes. Finally, the sections were mounted with neutral gum and observed using a fully motorized inverted fluorescence microscope (Ti2-E, Nikon, Japan).

### Nissl staining

A commercial kit (Nissl staining reagent, catalog no. G1436, Solarbio, China) was used to observe the Nissl body of surviving neurons in the spinal cord and brain on the 21^st^ day post-SCI. According to the manufacturer's instructions, the spinal cord and brain sections were immersed in the Nissl staining reagent for 30 minutes at 55°C, followed by rinsing with ultrapure water. The sections were subsequently dehydrated using graded ethanol solutions (80%, 90%, 95%, and 100%), and images were acquired using a fully motorized inverted fluorescence microscope (Ti2-E, Nikon, Japan).

### Basso, Beattie and Bresnahan (BBB) scale

Neurological function was evaluated using the BBB scale at 0, 3, 7, 14, and 21 days after SCI, following the methods of Basso *et al.* 26. The BBB scale is commonly used to assess hindlimb locomotor function in rats, with scores ranging from 0 (maximal deficits) to 21 (normal function). Prior to the formal test, the rats were placed in an open field (1 m × 1 m) for 5 minutes to acclimate to the test environment. Each rat was assessed by two independent examiners who were blinded to the treatment regimen.

### Modified Rivlin inclined plate test

To further assess functional recovery following rTSMS treatment, a modified Rivlin inclined plate test was conducted at 21 days post-SCI, following the method described in previous research 27. Briefly, the rats were placed on the inclined plate for 5 minutes prior to testing. The incline angle of the plate was then increased by 5° for each trial, and the maximum angle (held for 5 seconds) was recorded. Each rat underwent five trials, and the examination was conducted by two independent observers who were blinded to the experimental groups.

### Horizontal ladder test

The horizontal ladder test is a reliable and innovative method for assessing skilled walking and motor coordination 28, 29. Briefly, the rats were placed on a horizontal ladder apparatus (60 cm in length) for 5 minutes to acclimate to the environment. Each test session was recorded using a camera. Positive events in the horizontal ladder test included plantar steps, toe steps, and skips, whereas negative events consisted of slips, misses, and drags. An increased number of positive events indicated improved motor function recovery in SCI rats. A ladder beam score (LBS) was calculated using the following formula: LBS% = number of positive events / total number of events × 100%.

### Heat pain assessment

To assess sensory function in rats with SCI, a Heat-Induced Pain Detector PL-200 (TECHMAN SOFT, China) was utilized to evaluate hind paw sensitivity to photothermal stimulation 30. On the 21^st^ post-operative day, all rats were allowed to acclimate to the environment for 15 minutes prior to the formal test. A heat source radiator set at 50% intensity was then used to stimulate the animal's paw, and the paw withdrawal latency was recorded and analyzed.

### Motor-evoked potentials (MEPs) assessment

To gather additional electrophysiological data, we used a noninvasive method to detect MEPs in rats. As previously described, the CCY1 magnetic stimulation device was used to assess the MEPs of rats at 7, 14, and 21 days after SCI. Briefly, the center of the stimulation coil was positioned above the center of the skull, and stimulation was delivered at 100% maximum intensity. MEPs were recorded from the tibialis anterior muscle of the hindlimb in response to motor cortex stimulation. Each animal received 20 stimulations (with 30-second intervals), and the amplitude and latency of MEPs were recorded 31.

### Gait analyses

To quantify locomotor recovery after treatment, gait dynamics were assessed using the VisuGait analysis system (XINRUAN, Shanghai, China). Two previous studies were referenced for methodological guidance 32, 33. The walkway (60 cm long) was illuminated with uniform green fluorescence, while the overhead area emitted red light. The green-lit glass chamber enhanced footprint extraction during locomotion, and the red background facilitated the capture of the animal's contour. Image data was recorded with a high-speed camera connected to VisuGait software via a USB 3.0 interface. Before behavioral testing, all rats underwent a 7-day adaptation period in the experimental environment. On day 21 post-injury, gait parameters were evaluated for each group using the VisuGait analysis system. Measured parameters include regularity index, left hindlimb (LH) stance duration, right hindlimb (RH) stance duration, LH print position, and RH print position.

### Immunofluorescence staining

Frozen sections or cultured cells were subjected to immunofluorescence staining as follows. Slice samples from all groups or cultured cells were washed with PBS for 15 minutes, followed by permeabilization with 0.1% Triton X-100 for an additional 15 minutes. Next, the samples were incubated with 10% normal goat serum for 60 minutes at room temperature, followed by overnight incubation with primary antibodies at 4°C. Immunofluorescence staining was performed using primary antibodies against Neurofilament-200 (NF200, catalog no. ab134459, 1:500, Abcam, UK), Synapsin I (SYN, catalog no. AF6201, 1:500, Affinity Biosciences, USA), Calcitonin gene-related peptide (CGRP, catalog no. ab81887, 1:100, Abcam, UK), Tyrosine hydroxylase (TH, catalog no. MAB318, 1:500, Millipore, USA), IBA1 (catalog no. YM4765, 1:200, Immunoway, USA), CD68 (catalog no. ab283654, 1:100, Abcam, UK), iNOS (catalog no. NB300-605, 1:50, Novusbio, USA), NEUN (catalog no. ab177487, 1:200, Abcam, UK), cFOS (catalog no. ab302667, 1:200, Abcam, UK), Myelin basic protein (MBP, catalog no. 10458-1-AP, 1:200, ProteinTech, USA), cGAS (catalog no.55482, 1:200, SAB, USA), and STING (catalog no. 19851-a-AP, 1:200, ProteinTech, USA). After washing with PBS, the sections and cultured cells were incubated with fluorescein-conjugated secondary antibodies for 60 minutes in the dark at room temperature. Finally, the samples were counterstained with mounting medium containing 4',6-diamidino-2-phenylindole (DAPI, catalog no. ab104139, Abcam, UK), and images were observed under a laser scanning confocal microscope (Nikon AX, Japan).

### Western blotting

Following anesthetization and cardiac perfusion, spinal cord tissue (T9-T11) was promptly collected. First, the spinal cord tissues and cultured cells were homogenized using radioimmunoprecipitation assay (RIPA) lysis buffer (catalog no. P0013C, Beyotime, China), which contained a protease and phosphatase inhibitor cocktail (catalog no. P0013BP1051, Beyotime, China). The homogenate was then centrifuged for 20 minutes at 12,000 × g at 4°C to collect the supernatant. A commercial kit (Bicinchoninic Acid Kit, catalog no. 23227, Thermo Fisher, USA) was subsequently used to quantify the protein concentration.

Western blotting analysis was conducted as follows. First, proteins obtained from samples were separated using SDS-PAGE gels (catalog no. G2043, Servicebio, China) and subsequently transferred to polyvinylidene difluoride (PVDF) membranes (catalog no. IPVH00010, Merck Millipore, Germany) under constant current. Next, the PVDF membranes were incubated in blocking buffer (Tris-buffered saline containing 5% milk) for 60 minutes at room temperature, followed by an overnight incubation with primary antibody solutions at 4°C. The primary antibodies used included those against Postsynaptic density protein 95 (PSD95, catalog no. PSD95, 1:2000, Affinity Biosciences, USA), SYN (catalog no. AF6201, 1:2000, Affinity Biosciences, USA), NF200 (catalog no. ab134459, 1:2000, Abcam, UK), CD68 (catalog no. ab283654, 1:1000, Abcam, UK), iNOS (catalog no. NB300-605, 1:800, Novusbio, USA), IRF3 (catalog no. YT2398, 1:1000, Immunoway, USA), p-IRF3 (catalog no. YP0880, 1:1000, Immunoway, USA), NF-kB-P65 (P65, catalog no. YT3108, 1:1000, Immunoway, USA), p-NF-kB-P65 (p-P65, catalog no. AF2006, 1:1000, Affinity Biosciences, USA), cGAS (catalog no. 55482, 1:1000, SAB, USA), STING (catalog no. 19851-a-AP, 1:1000, ProteinTech, USA), GAPDH (catalog no. ab8245, 1:1000, Abcam, UK), and β-actin (catalog no. ab8226, 1:1000, Abcam, UK). The following day, after being washed three times with Tris-buffered saline with Tween 20 (TBST), the PVDF membranes were incubated with the corresponding secondary antibodies for 60 minutes at room temperature. Finally, an enhanced chemiluminescence kit (catalog no. A38555, Thermo Fisher, USA) was used to detect the immunoreactive bands.

### Sample preparation for single-cell RNA sequencing (sc-RNA-seq)

Spinal cord tissue samples from the control and low-frequency rTSMS groups were collected on the 21^st^ day post-SCI. First, the spinal cord tissue was washed with PBS. The tissue sample was then enzymatically digested to obtain a single-cell suspension, which was terminated with complete medium (Dulbecco's modified Eagle's medium + 10% fetal bovine serum). Next, the suspension was filtered through 70-µm (catalog no. 258368-1, Nest, China) and 40-µm (catalog no. 258369-1, Nest, China) cell strainers. Finally, the pellet was resuspended in PBS, and the cell density and viability were assessed using a BD Rhapsody™ Scanner (BD Biosciences, La Jolla, CA, USA).

### Single-cell transcriptome capture, library construction, and sequencing

All libraries for sc-RNA-seq were generated as previously described 34. In brief, cell capture was achieved by randomly distributing a single-cell suspension into microwells. Oligonucleotide barcodes were generated to ensure that each bead paired with each cell. The cells were lysed in the microwells, allowing the mRNA molecules to hybridize to the beads. The beads were then collected into a single tube for cDNA synthesis. Each cDNA molecule was subsequently labeled at the 5' end, corresponding to the 3' end of the mRNA transcript, with a unique molecular identifier (UMI) and cell labeling information. Sequencing for each sample was conducted using an Illumina sequencer (Illumina, San Diego, CA) with a 150-bp paired-end run.

### Raw data analysis and functional annotation

Quality control, dimensionality reduction, and clustering were conducted using the Seurat package version 4.3.1 35, whereas batch correction was performed using the Harmony package 36. Cells with fewer than 200 detected genes or more than 15% mitochondrial gene content were excluded. Highly variable genes were identified, and principal component analysis (PCA) was performed with Seurat. Batch effects across samples were mitigated using Harmony with the parameter max.iter.harmony set to 20. Clustering analysis was carried out using the FindNeighbors and FindClusters functions with a resolution of 0.2. The top 30 Harmony components were used for dimensionality reduction and visualization via t-SNE.

The FindMarkers function in Seurat was applied to identify genes with significant differential expression between distinct cell populations. Differentially expressed genes (DEGs) were defined using the thresholds of adjusted *p*-value < 0.05 and absolute average log2 fold change > 1. Enriched pathways were identified by querying the Gene Ontology (GO), Kyoto Encyclopedia of Genes and Genomes (KEGG), and Reactome databases 37-39. Functional enrichment analysis was conducted using the clusterProfiler package 40. Differentially expressed genes were ranked on the basis of their average log fold change and used for downstream analysis. Significantly enriched gene sets were interpreted within the context of established biological processes and pathways. The gene set enrichment analysis (GSEA) results were visualized using the clusterProfiler package in R 41.

### Quantitative real-time PCR (qRT-PCR)

Total RNA was isolated from spinal cord tissues using TRIzol reagent (catalog no. 15596026, Invitrogen, USA) following the manufacturer's protocol. The purified RNA was reverse transcribed into cDNA using a reverse transcription reagent (catalog no. AG11706, AG, China). Quantitative PCR was performed using qPCR-premixed solution (catalog no. A301-05, GenStar, China) on a CFX Connect Real-Time PCR System (Bio-Rad, USA). Gene expression levels were normalized to* Gapdh* and analyzed using the 2^ (-ΔΔCt) method. All gene-specific primers were designed and synthesized by Sangon Biotech (Shanghai, China), with sequences as follows: *IL-1β* forward, CTCACAGCAGCA TCTCGACAAGAG; *IL-1β* reverse, TCCACGGGCAAGACATA GGTAGC;* IL-6* forward, ACTTCCAGCCAGTTGCCTTCT TG; *IL-6* reverse, TGGTCTGTTGTGGGTGGTATCCTC; *TNFa* forward, ATGGTCTCATCAGTTCC; *TNFa* reverse, GCTCCTCCGCTTGGTGGTTTG; *Gapdh* forward, ACGGCAAGTTCAACGGCACAG; *Gapdh* reverse, CGACATACTCAGCACCAGCATCAC.

### Primary microglia culture

Primary cortical microglial cultures were established following a previously described method 42. Briefly, the cortices of postnatal day 2 (P2) SD rat pups were dissected with sterile scissors. The tissues were then mechanically dissociated and digested with 0.125% trypsin (catalog no. T1300, Solarbio, China) at 37 °C for 15 minutes. The cells were subsequently seeded in plates coated with 0.1 mg/ml poly-D-lysine (PDL, catalog no. ST508, Beyotime, China) and maintained in minimum essential medium (MEM, catalog no. C11095500BT, Gibco, USA) supplemented with 10% fetal bovine serum (FBS, catalog no. SFBS, BOVOGEN, Australia) and 1% penicillin/streptomycin (catalog no. 15140122, Gibco, USA) at 37 °C in a 5% CO_2_ atmosphere for 10 days. Subsequently, the cultures were shaken at 180 rpm for 2 hours at 37 °C to collect and purify the microglia.

### Grouping and magnetic stimulation (MS) treatment of microglia *in vitro*

To simulate the SCI-induced inflammatory environment *in vitro*, microglia were exposed to lipopolysaccharide (LPS), as previously described 43. For our pilot experiment, microglia were cultured in 24-well plates at a density of 1 × 10^5^ cells/well for 12 hours. After 24 hours of LPS treatment (1 µg/ml, catalog no. L2880-25MG, Sigma-Aldrich, USA), the cells were treated with or without magnetic stimulation 44.

Microglia were randomly assigned to four groups. (1) In the normal (Nor) group, the cells were cultured in normal medium without LPS treatment; (2) In the control (Ctrl) group, the cells were cultured in normal medium for 12 hours, followed by LPS treatment and sham magnetic stimulation for 2 days; (3) In the MS group, the cells were treated with LPS and then subjected to magnetic stimulation treatment once a day for 2 days at a frequency of 1 Hz; and (4) In the 2,3 cGAMP group, the cells were treated with LPS and then with 2,3 cGAMP (catalog no. L2880-25MG, Sigma-Aldrich, USA) at a concentration of 20 µM for 6 hours 45, followed by magnetic stimulation for 2 days.

### Synaptosomes purification

Synaptosomal fractions were isolated following established protocols 46, 47. In brief, brain tissues were extracted and homogenized in sucrose solution (0.32 M sucrose, 25 mM HEPES, with protease inhibitors). The homogenate was centrifuged (4 °C, 1400 × g, 10 minutes) to obtain supernatant S1 (total lysate) from nuclear material and cellular debris. S1 was subsequently centrifuged (4 °C, 10,000 × g, 12 minutes) to yield P2 (crude synaptosomes). P2 was washed with sucrose buffer and resuspended in cold HBS solution (25 mM HEPES, 150 mM NaCl, with protease inhibitors) to generate synaptosomes.

### Engulfment assays

According to established methodology 47, 48, synaptosomes were labeled with pHrodo Red dye (Catalog no. P36600, Thermo Fisher, USA) at 25 °C for 2 hours. Subsequently, labeled synaptosomes were obtained via sequential centrifugations to remove unbound dye. For phagocytosis assays, microglia were incubated with pHrodo Red-conjugated synaptosomes for 2 hours. Non-internalized substrate was eliminated by PBS washing, and then the microglia proceeded to the next experiment.

### Statistical analysis

All the statistical analyses were conducted using GraphPad Prism 9.0 software (GraphPad Software Inc., US), and the data were presented as the means ± standard deviations. Data obtained from the BBB and MEPs test were analyzed using repeated-measures analysis of variance (ANOVA). For comparisons among three or more independent groups (HE staining, Nissl staining, modified Rivlin inclined plate test, horizontal ladder test, thermal pain assessment test, gait test, western blotting, and immunofluorescence staining), one-way ANOVA with Tukey's multiple comparison test was employed. The student's t test was used for statistical analysis of comparisons between two independent groups. A *p* value < 0.05 was considered to indicate statistical significance.

## Results

### Low-frequency rTSMS attenuates lesion area and neuron damage after SCI

To determine the optimal frequency for rTSMS initiated in the sub-acute phase after SCI, the experimental design was illustrated in Fig. 1E. The integrity of nerve tissue and neuron survival in the lesion area are critical for improving neurological function. Therefore, HE and Nissl staining were performed to assess the effects of rTSMS on structural recovery. Longitudinal section of the spinal cord was presented in Figure S1C. HE staining revealed a significant cavity at the lesion site following SCI (Ctrl vs. Sham, *P* < 0.001). Importantly, treatment with 1 Hz rTSMS significantly reduced the lesion volume at 21 days post-injury compared with that in the Ctrl, 10 Hz, and 1+10 Hz groups. The statistical comparisons were as follows: 1 Hz vs. Ctrl, *P* < 0.001; 1 Hz vs. 10 Hz, *P* < 0.001; and 1 Hz vs. 1+10 Hz, *P* < 0.001 (Fig. 1F-G).

Concurrently, Nissl staining was performed to validate the effects of rTSMS on surviving neurons after SCI. Our findings revealed a significant loss of Nissl body at 21 days in rats with SCI (Ctrl vs. Sham, *P* < 0.001). However, following the 1 Hz rTSMS treatment, the quantity of Nissl body significantly increased (1 Hz vs. Ctrl, *P_(Rostral)_* = 0.0074,* P_(Caudal)_* = 0.0076). Moreover, compared with those in the 10 Hz and 1+10 Hz rTSMS groups, the Nissl body in surviving neurons were more abundant in the 1 Hz rTSMS group (1 Hz vs. 10 Hz, *P_(Rostral)_* = 0.0069,* P_(Caudal)_* = 0.0072; 1 Hz vs. 1+10 Hz,* P_(Rostral)_* = 0.0075,* P_(Caudal)_* = 0.0076; Fig. 1H-I). In summary, these histological results suggested that low-frequency rTSMS treatment initiated in the sub-acute phase significantly reverses SCI-induced damage to nerve tissue.

### Low-frequency rTSMS promotes functional recovery after SCI

**Figure 2:**
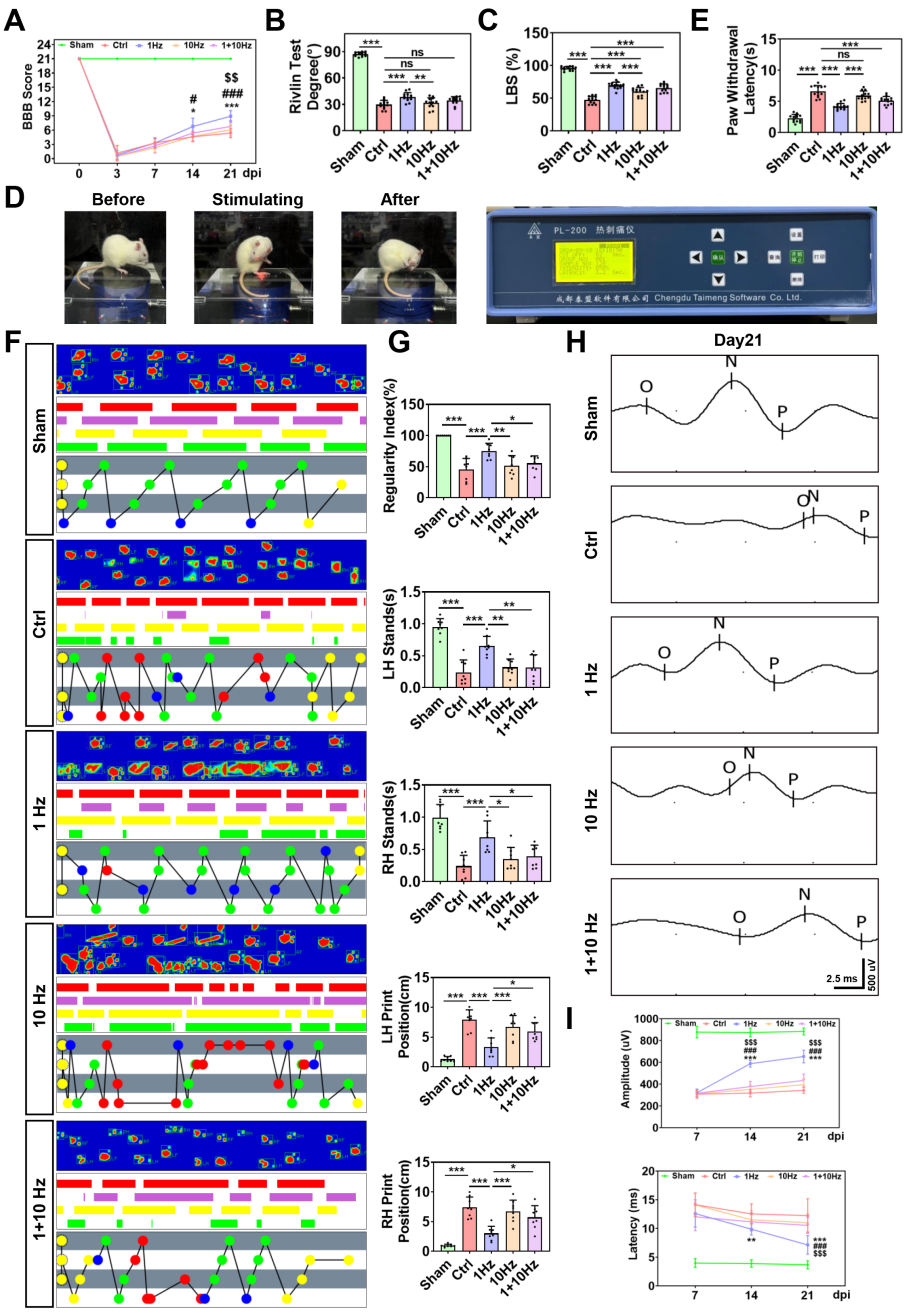
** Low-frequency rTSMS enhances motor and sensory function recovery following SCI.** (A) BBB scores of the rats at 0, 3, 7, 14, and 21 days after SCI, n = 13. (B) Rivlin test scores of the rats at 21 dpi, n = 13. (C) Ladder test scores of the rats at 21 dpi, n = 13. (D) Representative images obtained before and after photothermal stimulation. (E) Statistical analysis of paw withdrawal latency in response to photothermal stimulation at 21 dpi, n = 13. (F) Representative images of footprint pressure thermogram, footprints, and gait diagram. Footprint pressure thermogram: left forelimb (LF), left hindlimb (LH), right forelimb (RF), right hindlimb (RH); Footprint tracks with color coding: LF (red), LH (purple), RF (yellow), RH (green); Gait diagram: yellow circle (not take into consideration), blue circle (start point of sequence), green circle (normal sequence), red circle (abnormal sequence). (G) statistical analysis of gait parameters at 21 dpi, n = 8. (H-I) Representative individual electrophysiological traces and statistical analysis of the amplitude and latency of MEPs at 7, 14 and 21 dpi, n = 10. ^*^P < 0.05, ^**^P < 0.01, ^***^P < 0.001, 1 Hz group vs. Ctrl group;^ #^*P* < 0.05, ^##^*P* < 0.01,^ ###^*P* < 0.001, 1 Hz group vs. 10 Hz group; ^$^*P* < 0.05, ^$$^*P* < 0.01,^ $$$^*P* < 0.001, 1 Hz group vs. 1+10 Hz group.

To assess the effects of rTSMS on functional recovery, locomotor function of the hind limbs was evaluated using the BBB score test immediately prior to injury and at 3, 7, 14, and 21 days after SCI. All the SCI rats exhibited lower BBB scores at 3 days post-injury (dpi) compared to those in the Sham group. On the 14^th^ and 21^st^ days post-SCI, the BBB scores of the rats in the 1 Hz rTSMS group were greater than those in the Ctrl group (Day 14: 1 Hz vs. Ctrl, *P* = 0.0108; Day 21: 1 Hz vs. Ctrl, *P* < 0.001) and the 10 Hz rTSMS group (Day 14: 1 Hz vs. 10 Hz, *P* = 0.0163; Day 21: 1 Hz vs. 10 Hz, *P* < 0.001). Furthermore, compared with the 1+10 Hz rTSMS treatment, the 1 Hz rTSMS treatment significantly increased the BBB score at 21 dpi (1 Hz vs. 1+10 Hz, *P* = 0.0014). On the 21^st^ day post-SCI, the BBB score was significantly greater in the 1+10 Hz rTSMS group than in the Ctrl group (1+10 Hz vs. Ctrl, *P* = 0.0413), whereas the 10 Hz rTSMS did not significantly increase the BBB score (Fig. 2A). Additionally, the Rivlin test indicated that animals in the 1 Hz rTSMS group exhibited significant functional recovery compared with those in the 10 Hz rTSMS group (1 Hz vs. 10 Hz, *P* = 0.0043; Fig. 2B). The ladder test was also employed to evaluate behavioral performance following SCI. Images of the rats that underwent the ladder test before and after SCI surgery were provided in Figure S2A. Significant improvement in motor function was observed in the 1 Hz rTSMS group compared with both the Ctrl (1 Hz vs. Ctrl, *P* < 0.001) and 10 Hz rTSMS groups (1 Hz vs. 10 Hz, *P* < 0.001) at 21 dpi (Fig. 2C). Furthermore, sensory function was assessed using the plantar test. Compared with the Ctrl and 10 Hz rTSMS groups, the 1 Hz group exhibited a markedly reduced paw withdrawal latency at 21 dpi (1 Hz vs. Ctrl, *P* < 0.001; 1 Hz vs. 10 Hz, *P* < 0.001; Fig. 2D-E).

To further confirm the efficacy of rTSMS in treating SCI, gait analyses and electrophysiological test were conducted. Gait analyses data showed that the regularity index and the stance duration of animals in the 1 Hz rTSMS group were greater than that of the animals in the Ctrl, 10 Hz, and 1+10 Hz groups. On the contrary, 1 Hz rTSMS significantly reduced the print position compared to the Ctrl, 10 Hz, and 1+10 Hz groups (Fig. 2F-G). Additionally, compared with those in the Ctrl group, neural circuit activation in the Sham group was associated with greater MEPs amplitude and shorter MEPs latency. Greater MEPs amplitude was observed in the 1 Hz rTSMS group compared to other SCI model groups at 14 and 21 dpi (14 dpi: 1 Hz vs. Ctrl, *P* < 0.001; 1 Hz vs. 10 Hz, *P* < 0.001; 1 Hz vs. 1+10 Hz, *P* < 0.001; 21 dpi: 1 Hz vs. Ctrl, *P* < 0.001; 1 Hz vs. 10 Hz, *P* < 0.001; 1 Hz vs. 1+10 Hz, *P* < 0.001). Further, we found that 1 Hz rTSMS treatment significantly decreased the response latency of MEPs compared with those in the Ctrl, 10 Hz and 1+10 Hz groups at 21 dpi (1 Hz vs. Ctrl, *P* < 0.001; 1 Hz vs. 10 Hz, *P* < 0.001; 1 Hz vs. 1+10 Hz, *P* < 0.001; Figure S2B; Fig. 2H-I). Collectively, these results demonstrated that low-frequency rTSMS, initiated in the sub-acute phase after SCI, significantly accelerates neurological functional recovery compared to high-frequency rTSMS. Thus, a low-frequency rTSMS was employed in subsequent studies to explore the underlying mechanisms.

### rTSMS improves synapse reservation and neuronal activity after SCI

Emerging evidence demonstrates that the restoration of neurological functions is critically dependent on the quantity of neurons and the structural integrity of neurons 49, 50. Immunofluorescence staining data revealed that the number of neurons (NEUN^+^ positive cells) in the injured area significantly increased following rTSMS treatment (rTSMS vs. Ctrl, *P* < 0.001; Figure S3A-B). Additionally, double immunofluorescence staining for NF200 and MBP was conducted to evaluate the formation of myelinated axons. Compared with that in the Ctrl group, a significantly greater number of neural filaments were observed in the rTSMS group (NF200 intensity: rTSMS vs. Ctrl, *P* < 0.001; MBP intensity: rTSMS vs. Ctrl, *P* = 0.0170), with many of these regenerating filaments tightly wrapped by the myelin sheath (Figure S3C-D).

**Figure 3:**
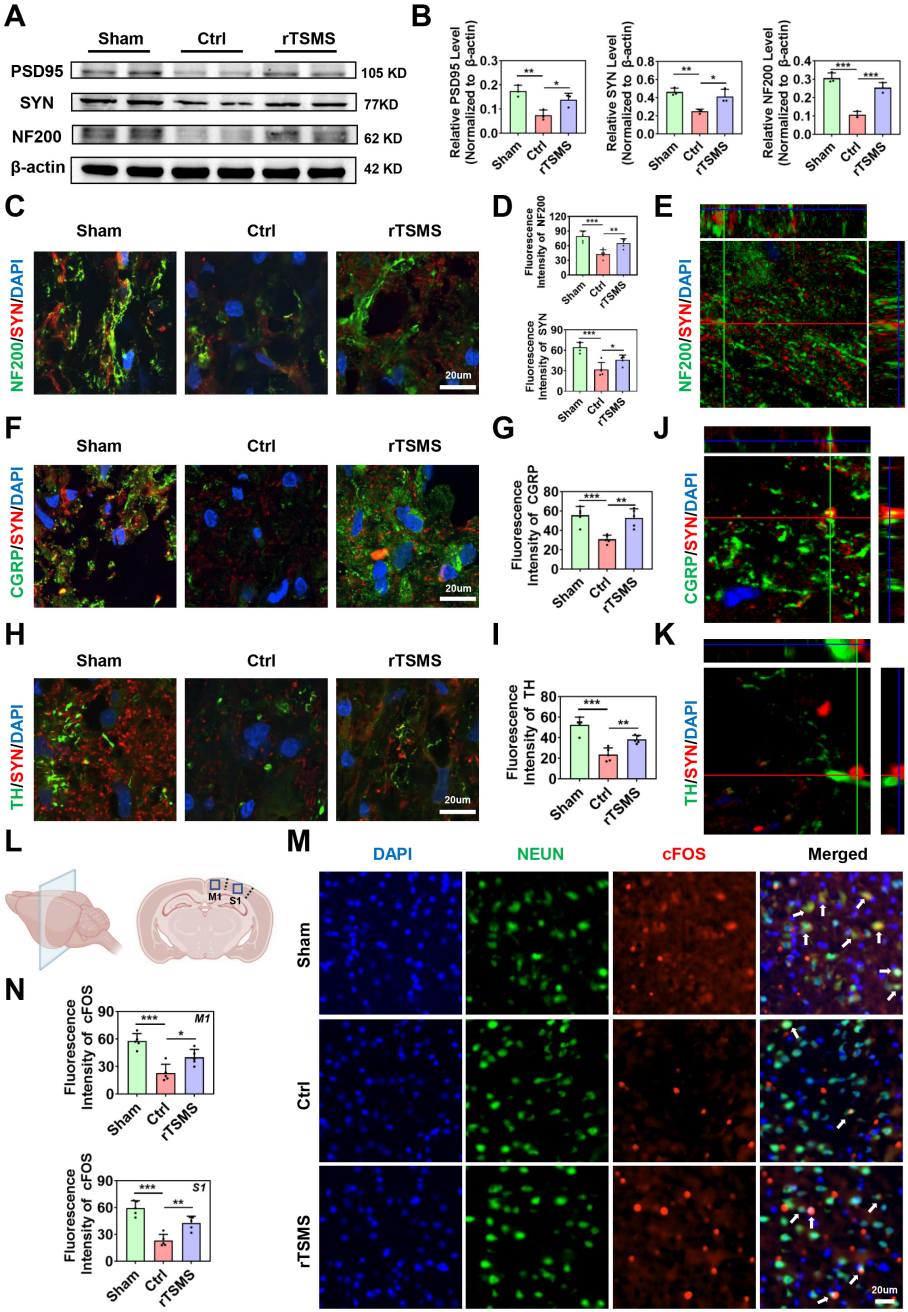
** rTSMS enhances synapse formation and neuronal activity in rats with SCI.** (A-B) Representative images of western blotting and quantitative analysis of PSD95, SYN and NF200 in the injured spinal cord at 21 dpi; n = 3. (C-D) Representative images of immunostaining and statistical analysis of NF200 (green), SYN (red) and DAPI (blue) in the injured spinal cord at 21 dpi; n = 5. (E) Z-stack image of NF200/SYN in the rTSMS group, slice thickness = 10 um, number of layers = 10. (F-G) Representative images of immunostaining and statistical analysis of CGRP (green), SYN (red) and DAPI (blue) in the injured spinal cord at 21 dpi, n = 5. (H-I) Representative images of immunostaining and statistical analysis of TH (green), SYN (red) and DAPI (blue) in the injured spinal cord at 21 dpi, n = 5. (J-K) Z-stack images of CGRP/SYN and TH/SYN in the rTSMS group, slice thickness = 10 um, number of layers = 10. (L) A schematic illustration showing the M1 and S1 regions in the brain. The image was generated with BioRender.com. (M) Representative immunostaining images of NEUN (green), cFOS (red) and DAPI (blue) in the S1 region at 21 dpi. (N) Statistical analysis of cFOS intensity in the M1 and S1 regions at 21 dpi, n = 5. *P < 0.05, **P < 0.01, ***P < 0.001.

To determine whether these regenerating axons form new synapses, we analyzed the expression levels of synapse-related markers using western blotting and immunofluorescence staining. Western blotting data demonstrated that the expression levels of PSD95, SYN, and NF200 were significantly lower in the Ctrl group than in the Sham group (PSD95: *P* = 0.0058; SYN: *P* = 0.0070; NF200: *P* < 0.001). However, these protein expression levels were increased following rTSMS treatment (PSD95: *P* = 0.0429; SYN: *P* = 0.0239; NF200: *P* < 0.001; Fig. 3A-B). Additionally, the immunofluorescence data revealed increased expression of NF200 and SYN in the rTSMS group compared with the Ctrl group (NF200: *P* = 0.0091; SYN: *P* = 0.0414; Fig. 3C-D). Co-localization analysis using Z-stack images revealed a close co-association between NF200 and SYN (Fig. 3E). Furthermore, compared with the Ctrl group, rTSMS treatment promoted sensory and motor axon regeneration, as shown by immunofluorescence staining for CGRP and TH, respectively (CGRP: *P* = 0.0025; TH:* P* = 0.0064; Fig. 3F-I). Importantly, sensory and motor axons formed synaptic structures (Fig. 3J-K).

Recently, it has been reported that spinal cord stimulation modulates the ascending and descending circuits and then influences brain function 51. In order to determine whether rTSMS affects the related brain areas, we evaluated the neuronal activity in primary motor cortex (M1) and primary somatosensory cortex (S1) (Fig. 3L). Immunofluorescence staining data suggested that the expression levels of cFOS in M1 and S1 regions were downregulated in the Ctrl group compared to the Sham group (M1: *P* < 0.001; S1:* P* < 0.001). However, a significant increase in the expression of cFOS was observed in the rTSMS group compared to the Ctrl group (M1: *P* = 0.0239; S1:* P* = 0.0041; Fig. 3M-N). Meanwhile, the number of Nissl bodies in the M1 and S1 area were significantly increased following rTSMS treatment compared with those in the Ctrl group (M1: *P* < 0.001; S1:* P* < 0.001; Figure S3E-F). Taken together, these findings demonstrated that rTSMS not only promotes synapse formation and axonal regeneration in the spinal cord but also improves neuronal activity in the brain.

### rTSMS diminishes microglia and affects the morphological features of microglia after SCI

**Figure 4:**
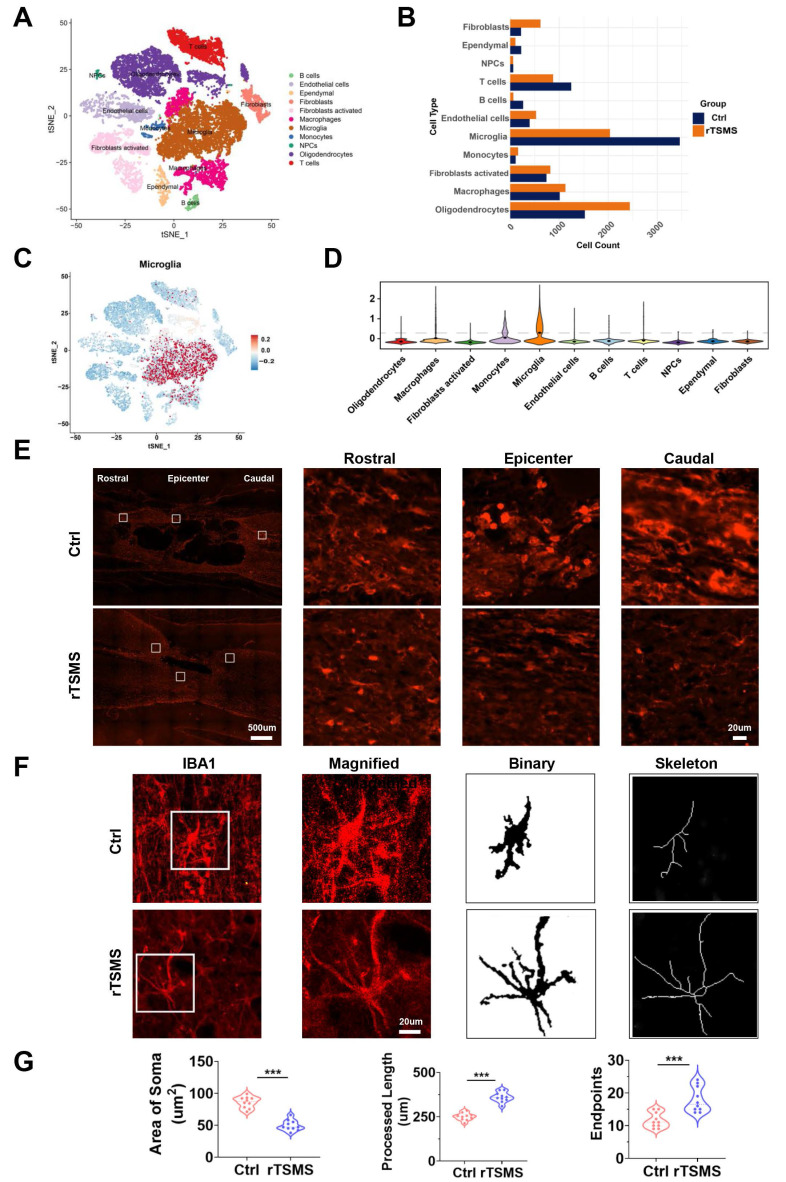
** rTSMS reduces the number of microglia and modulates the morphological features of microglia in SCI rats.** (A) Distribution characteristics of cell subpopulations in injured spinal cord tissue between the Ctrl and rTSMS groups. (B) Changes in the proportion of cells in the Ctrl and rTSMS groups. (C-D) Expression levels of microglial markers (*Cx3cr1*, *Aif1*, *P2ry12*, *Tmem119* and *Iba1*) across different cell subpopulations. (E) Representative images of IBA1 immunostaining (red) in the spinal cord at 21 dpi. (F) Representative images of IBA1 immunostaining (red) in the injured spinal cord and skeletonized analysis. (G) Quantification and morphology analysis of IBA1**^+^** microglia in the Ctrl and rTSMS groups. ***P < 0.001.

To explore the underlying mechanism of rTSMS-derived neuroprotection at the cellular level, spinal cord tissue was collected for sc-RNA-seq analysis. Eleven cell types were identified and visualized using t-SNE, including B cells, endothelial cells, ependymal cells, fibroblasts, activated fibroblasts, macrophages, microglia, monocytes, neural progenitor cells (NPCs), oligodendrocytes, and T cells (Fig. 4A). Changes in the proportions of these cell types between the rTSMS-treated and untreated groups were shown in Fig. 4B. In the Ctrl group, microglia accounted for the largest proportion of the cells. However, following rTSMS treatment, the relative proportion of microglia significantly decreased, suggesting that microglia were among the target cells actively responding to rTSMS treatment. Moreover, the expression of microglial cell markers (*Cx3cr1*, *Aif1*, *P2ry12*, *Tmem119*, and *Iba1*) across different cell subpopulations was assessed (Fig. 4C-D).

Importantly, consistent with the sc-RNA-seq results, the immunofluorescence staining results demonstrated that rTSMS treatment significantly decreased the number of microglia after SCI (Fig. 4E). We further analyzed the morphological changes in IBA1-positive microglia before and after treatment. After SCI, numerous microglia in the Ctrl group presented an enlarged cell body, decreased summed process length, and a reduced number of cellular processes. In contrast, after rTSMS treatment, microglia displayed a more ramified morphology (Fig. 4F-G). These results suggested that microglia might be a primary target of rTSMS treatment.

### rTSMS alleviates synapse loss by inhibiting neuroinflammation and the phagocytic activity of M1 microglia after SCI

**Figure 5:**
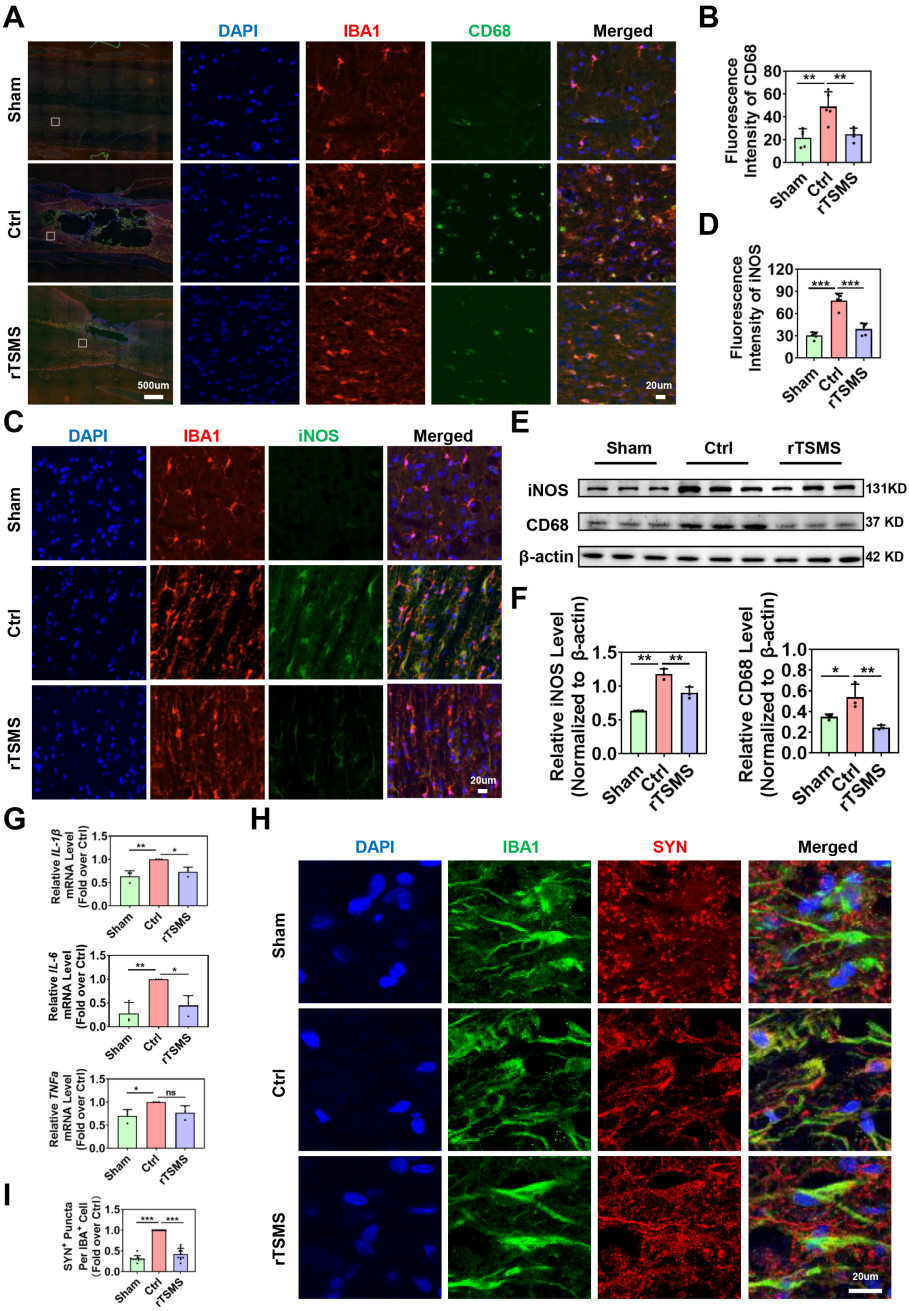
** rTSMS inhibits synapse loss by decreasing neuroinflammation and microglial phagocytosis after SCI.** (A-B) Representative immunostaining images and statistical analysis of IBA1 (red), CD68 (green), and DAPI (blue) in the spinal cord at 21 dpi, n = 5. (C-D) Representative immunostaining images and statistical analysis of IBA1 (red), iNOS (green), and DAPI (blue) in the center of the injured spinal cord at 21 dpi, n = 5. (E-F) Representative images of western blotting and quantitative analysis of CD68 and iNOS in injured spinal cord tissue at 21 dpi, n = 3. (G) mRNA expression of* IL-1β*, *IL-6*, and *TNFα* in each group at 21 dpi, n = 3. (H-I) Representative images of IBA1 (green), SYN (red), and DAPI (blue) immunostaining in the center of the injured spinal cord at 21 dpi. (I) Statistical analysis of puncta in microglia in each group. n = 10. *P < 0.05, **P < 0.01, ***P < 0.001.

Considering the central role of microglia in the harsh environments, we further investigated the effects of magnetic stimulation on microglial function. First, we assessed whether rTSMS affects M1 phenotype microglial activation by co-immunostaining IBA1 with CD68 or iNOS, which are considered markers of microglial pro-inflammatory activation 52. As shown in Fig. 5A-D, immunofluorescence staining revealed more CD68-positive microglia (CD68^+^IBA1^+^ cells) and iNOS-positive microglia (iNOS^+^IBA1^+^ cells) in the Ctrl group than in the Sham group (CD68 intensity: *P* = 0.0015; iNOS intensity: *P* < 0.001). However, rTSMS treatment significantly reversed the increase in the number of CD68-positive microglia and iNOS-positive microglia induced by SCI (CD68 intensity: *P* = 0.0037; iNOS intensity: *P* < 0.001). Furthermore, western blotting revealed that SCI significantly increased the protein levels of CD68 and iNOS (CD68 protein: Ctrl vs. Sham, *P* = 0.0496; iNOS protein: Ctrl vs. Sham, *P* = 0.0012). However, these effects were markedly reversed by rTSMS treatment (CD68 protein: rTSMS vs. Ctrl, *P* = 0.0077; iNOS protein: rTSMS vs. Ctrl, *P* = 0.0061; Fig. 5E-F). Additionally, our results indicated that rTSMS markedly reduced the mRNA expression of pro-inflammatory mediators (*IL-1β* and *IL-6*) at 21 days post-SCI (*IL-1β*: rTSMS vs. Ctrl, *P* = 0.0258; *IL-6*: rTSMS vs. Ctrl, *P* = 0.0221; Fig. 5G).

Recent studies have shown that microglia are positively involved in synaptic plasticity in the CNS 53, 54. Therefore, we next explored whether rTSMS alleviates synapse loss by inhibiting microglial overactivation. Immunofluorescence staining confirmed the notable colocalization of IBA1 and SYN in the injured spinal cord at 21 days after SCI. However, after consecutive rTSMS treatment, there was a significant reduction in synapse phagocytosis by microglia (rTSMS vs. Ctrl,* P* < 0.001; Fig. 5H-I). These findings collectively suggested that rTSMS alleviates the neuroinflammatory response and suppresses synapse phagocytosis by inhibiting M1 microglial overactivation.

### cGAS-STING plays an important role in the rTSMS-induced modulation of microglia

**Figure 6:**
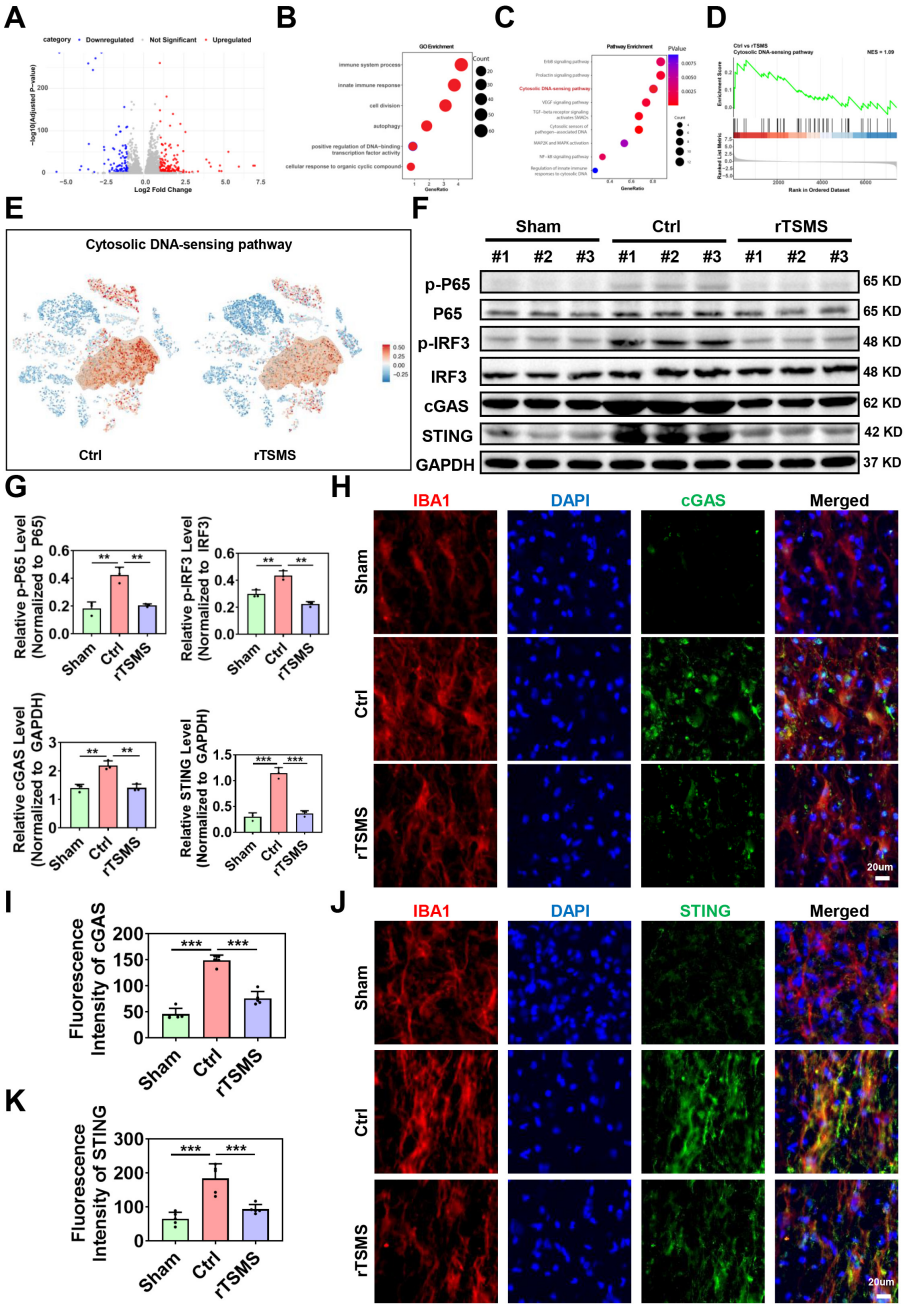
** The cGAS-STING pathway is crucial for rTSMS-induced microglial modulation.** (A) A volcano plot illustrating the genes differentially expressed in microglia following rTSMS treatment. Red and blue dots represented upregulated and downregulated genes, respectively. (B) GO analysis of downregulated genes in microglia following rTSMS treatment. (C) KEGG analysis of genes downregulated in microglia following rTSMS treatment. (D) GSEA indicated that the cytosolic DNA-sensing pathway was inhibited by rTSMS. (E) Visualization of cGAS-STING pathway activity on the t-SNE. Each point represented a single cell, colored by the cGAS-STING pathway activity score computed via module scoring of canonical pathway genes (e.g., *Cgas*, *Sting*, *Tbk1*, *Irf3*, *Nfkb1*, *Rela*, *Ikbkb*, *Chuk*, *Casp1*, *Ifnb1*). Warmer colors (red) indicated higher pathway activation, while cooler colors (blue) indicated lower activity. A semi-transparent contour highlighted a distinct cellular subpopulation exhibiting elevated cGAS-STING activity. (F-G) Representative images of western blotting and quantitative analyses of p-P65, p-IRF3, cGAS and STING in injured spinal cord tissue at 21 dpi, n = 3. (H-I) Representative immunostaining images and statistical analyses of IBA1 (red), cGAS (green), and DAPI (blue) in the center of the injured spinal cord at 21 dpi, n = 5. (J-K) Representative images and statistical analyses of IBA1 (red), STING (green), and DAPI (blue) immunostaining in the center of the injured spinal cord at 21 dpi, n = 5. **P < 0.01, ***P < 0.001.

To further investigate the molecular mechanisms underlying rTSMS-induced microglial modulation, a volcano plot was generated to illustrate the genes that were differentially expressed in microglia between the rTSMS-treated and untreated groups (Fig. 6A). GO analysis revealed functional annotations, including immune system processes, the innate immune response, cell division, autophagy, positive regulation of DNA-binding transcription factor activity, and the cellular response to organic cyclic compounds (Fig. 6B). Further KEGG analysis of genes which were downregulated in microglia following rTSMS revealed associations with several pathways, including the ErbB signaling pathway, prolactin signaling pathway, cytosolic DNA-sensing pathway, VEGF signaling pathway, TGF-beta receptor signaling activating SMADs, cytosolic sensors of pathogen-associated DNA, MAP2K and MAPK activation, NF-kB signaling pathway, and regulation of innate immune responses to cytosolic DNA (Fig. 6C). As shown in Fig. 6D-E, both GSEA and t-SNE analyses data indicated that the cytosolic DNA-sensing pathway was significantly inhibited in microglia after rTSMS treatment. This was supported by the downregulation of key pathway components (e.g., *Cgas*, *Sting*, *Tbk1*, *Irf3*, *Nfkb1*, *Rela*, *Ikbkb*, *Chuk*, *Casp1*, *Ifnb1*) in microglial clusters after rTSMS intervention, which providing direct sc-RNA-seq- based evidence for pathway inhibition at the cell-type level.

Emerging studies have demonstrated that cGAS is a critical cytosolic DNA sensor that activates innate immune responses through the production of the second messenger cGAMP, which in turn activates the adaptor STING 55. Importantly, recent studies have shown that the regulation of the cGAS-STING pathway of cytosolic DNA sensing plays a crucial role in SCI 56. On the basis of our sc-RNA-seq results, we speculated that the cGAS-STING pathway of cytosolic DNA sensing in microglia might be an important target of rTSMS treatment.

We subsequently verified whether the cGAS-STING signaling pathway is activated in microglia following SCI and whether rTSMS regulates this pathway. As presented in Fig. 6F-G, rats in the Ctrl group presented elevated protein expression levels of p-P65, p-IRF3, cGAS and STING compared with those in the Sham group (p-P65: *P* = 0.0011; p-IRF3: *P* = 0.0025; cGAS: *P* = 0.0012; STING: *P*<0.001). However, a significant reduction in the expression of p-P65, p-IRF3, cGAS and STING was observed in the rTSMS group compared with the Ctrl group (p-P65: *P* = 0.0018; p-IRF3: *P* = 0.0011; cGAS: *P* = 0.0012; STING: *P*<0.001).

We further examined the colocalization of cGAS and STING in microglia using double-label immunofluorescence staining. Co-immunostaining analyses indicated that rats in the Ctrl group exhibited a greater number of cGAS^+^IBA1^+^ positive cells compared to those in the Sham group (*P* < 0.001). In contrast, rats in the rTSMS group presented a decreased number of cGAS^+^IBA1^+^ positive cells compared with those in the Ctrl group (*P* < 0.001; Fig. 6H-I). Importantly, a similar result was observed with double immunofluorescence labeling of STING and IBA1 (Ctrl vs. Sham, *P* < 0.001; rTSMS vs. Ctrl, *P* < 0.001; Fig. 6J-K). Collectively, these data suggested that SCI activates the spinal cGAS-STING pathway and that rTSMS markedly inhibits the activation of this signaling pathway in microglia.

### A STING agonist weakens the effects of MS on microglial activation and phagocytosis *in vitro*

**Figure 7:**
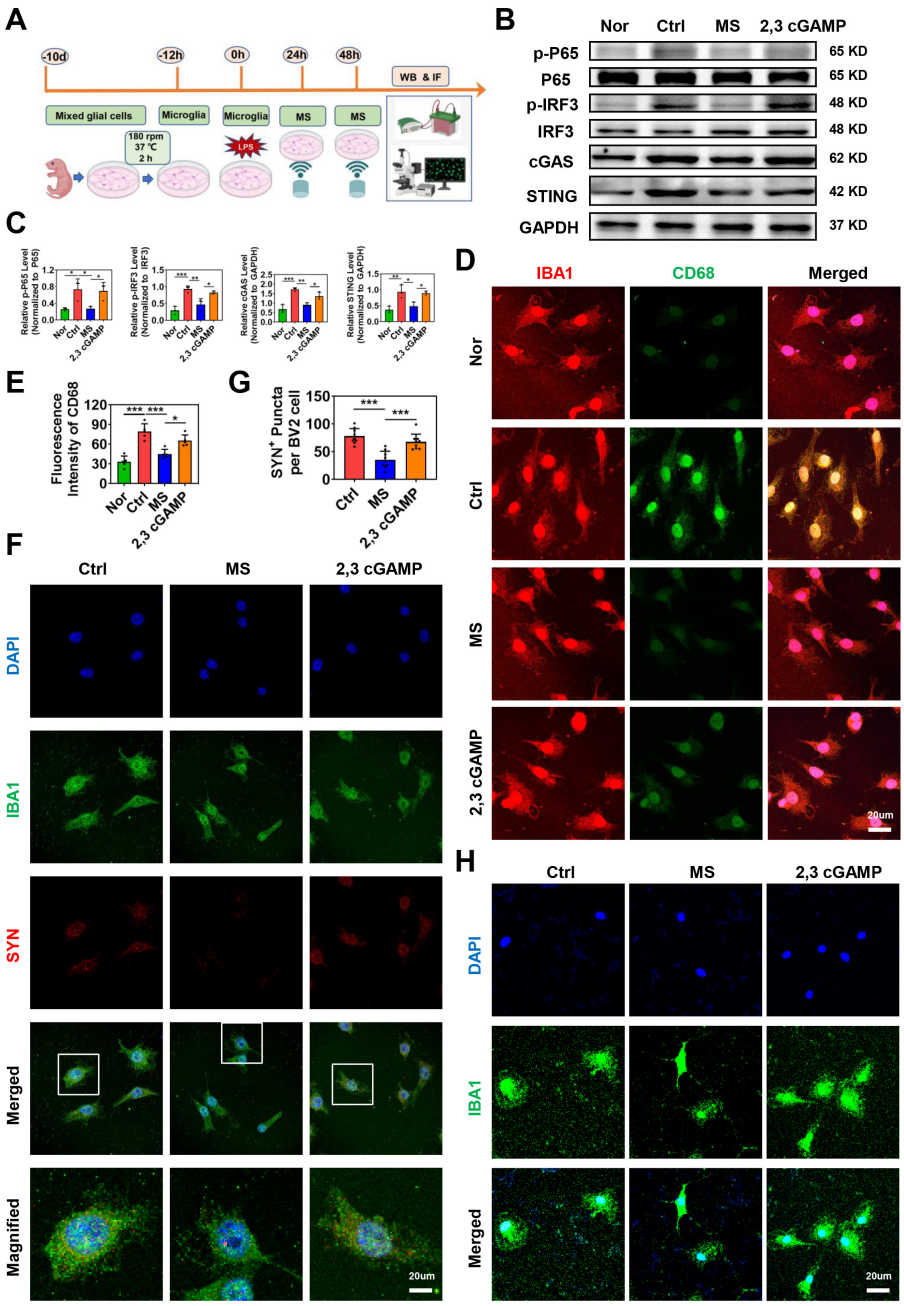
** STING agonist impairs the effects of MS on microglial activation and phagocytosis.** (A) Experimental design *in vitro*. The image was created with BioRender.com. (B-C) Representative images of western blotting and quantitative analyses of p-P65, p-IRF3, cGAS and STING in primary microglia, n = 3. (D-E) Representative immunostaining images and statistical analyses of IBA1 (red) and CD68 (green) in primary microglia. (F) Representative immunostaining images of the colocalization of synaptosomes (red) and Iba1^+^ microglia (green). (G) Statistical analysis of SYN^+^ puncta in BV2 microglia. n = 9. (H) Representative immunostaining images of IBA1 (green) and DAPI (blue) in primary microglia. *P < 0.05, **P < 0.01, ***P < 0.001.

To investigate whether MS affects microglial phagocytosis via the cGAS-STING signaling pathway, primary microglia were isolated and subjected to MS treatment (Fig. 7A). In addition, a STING agonist (2,3 cGAMP) was used for reverse intervention experiments. First, the expression levels of cGAS-STING pathway-related proteins were assessed using western blotting. Compared with those in the Nor group, the protein levels of p-P65, p-IRF3, cGAS and STING in primary microglia were significantly increased following LPS treatment (p-P65, *P* = 0.0281; p-IRF3, *P* < 0.001; cGAS, *P* < 0.001; STING, *P* = 0.0052). However, MS significantly inhibited the expression levels of p-P65, p-IRF3, cGAS and STING in microglia (p-P65, *P* = 0.0320; p-IRF3, *P* = 0.0065; cGAS, *P* = 0.0018; STING, *P* = 0.0181). In contrast, the addition of 2,3 cGAMP reversed the inhibitory effects of MS on microglia (p-P65, *P* = 0.0497; p-IRF3, *P* = 0.0277; cGAS, *P* = 0.0455; STING, *P* = 0.0301; Fig. 7B-C).

Moreover, immunofluorescence staining revealed many activated M1 microglia (CD68^+^IBA1^+^ cells) in the Ctrl group. Following MS intervention, the number of CD68^+^IBA1^+^ cells decreased compared with that in the Ctrl group. However, 2,3 cGAMP increased the number of CD68^+^IBA1^+^ cells compared with that in the MS group (Fig. 7D-E). Further, we found that MS markedly reduces microglia phagocytosis of synaptosomes while 2,3 cGAMP enhances phagocytosis activity of microglia *in vitro* (MS vs. Ctrl, *P* < 0.001; 2,3 cGAMP vs. MS, *P* < 0.001; Fig. 7F-G). Morphometric analysis further revealed that LPS treatment reduced both the process length and number of branches of microglia, suggesting that under LPS conditions, microglia possessed a stronger ability to phagocytose synapse. However, microglia in the MS treatment group presented a more ramified morphology, with an increased process length and more branches. Notably, 2,3 cGAMP diminished the effects of MS on microglia (Fig. 7H). In summary, these findings demonstrated that MS restores microglial morphology to a more ramified state under LPS conditions, which regulate the phagocytic function of microglia via the cGAS-STING pathway *in vitro*.

### STING agonist reverses the neuroprotective effects of rTSMS in rats with SCI

**Figure 8:**
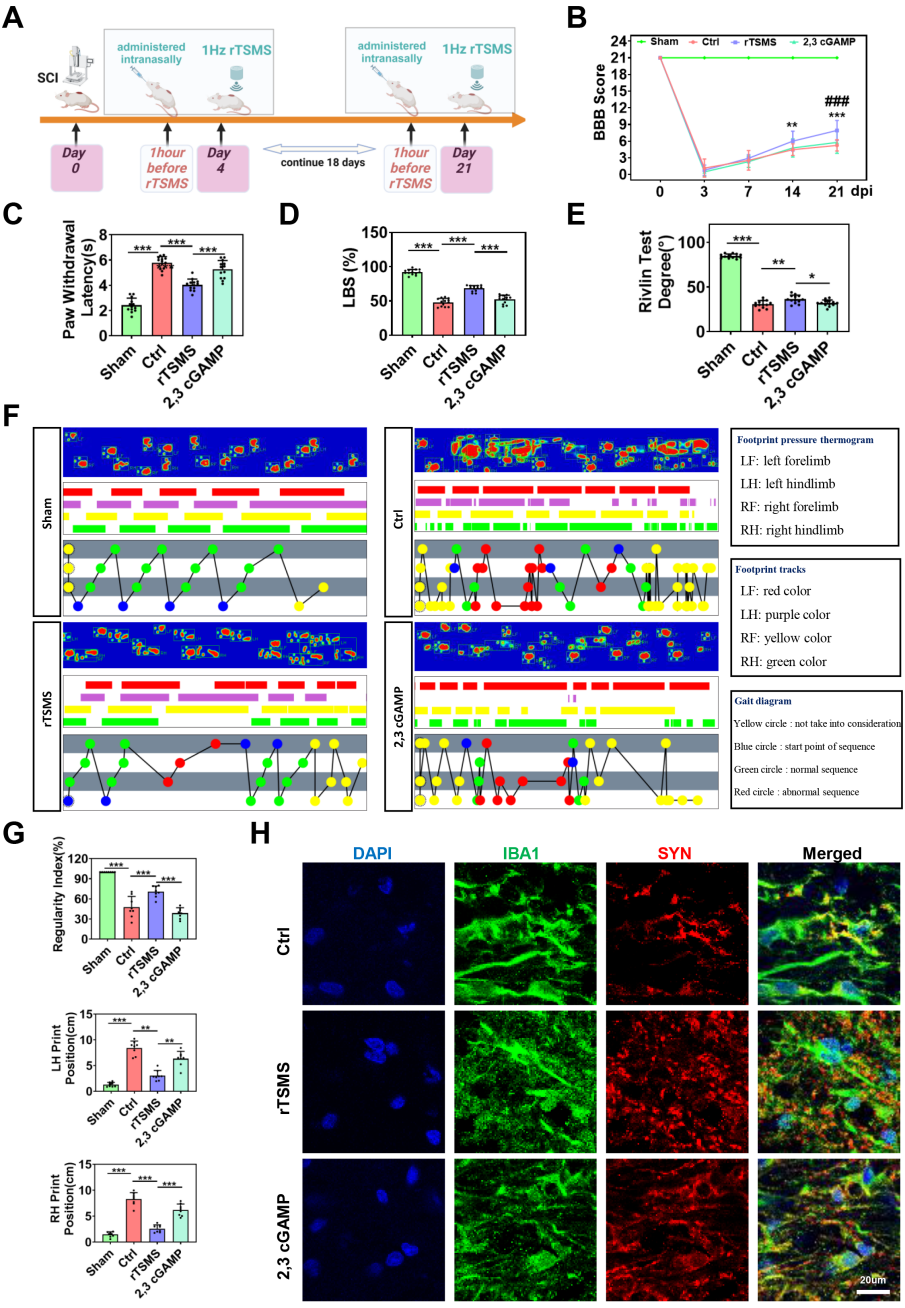
**STING agonist diminishes the neuroprotective effects of rTSMS in SCI rats.** (A) Experimental design. The image was created with BioRender.com. (B) BBB scores of the rats in each group at 0, 3, 7, 14, and 21 dpi, n = 13. (C) Statistical analysis of paw withdrawal latency in response to photothermal stimulation at 21 dpi, n = 13. (D) Ladder test scores of the rats at 21 dpi, n = 13. (E) Rivlin test scores of the rats at 21 dpi, n = 13. (F) Representative images of footprint pressure thermogram, footprints, and gait diagram. (G) Statistical analysis of gait parameters at 21 dpi, n = 8. (H) Representative immunostaining images of IBA1 (green), SYN (red), and DAPI (blue) at the injured site at 21 dpi. *P < 0.05, **P < 0.01, ***P < 0.001.

To further clarify the role of the cGAS-STING pathway in the neuroprotective effects of rTSMS, we also used a STING agonist (2,3 cGAMP) *in vivo*. The experimental design was presented in Fig. 8A. Initially, histological changes were assessed by HE staining, which revealed a reduction in lesion size in rats subjected to rTSMS treatment. However, this effect was significantly reversed by 2,3 cGAMP (*P* < 0.001; Figure S4A-C). Additionally, the number of Nissl bodies near the lesion site was evaluated by Nissl staining. Compared with that in the Ctrl group, the number of Nissl bodies significantly increased following rTSMS treatment, whereas the 2,3 cGAMP group presented a decreased number of Nissl bodies compared with the rTSMS group (*P_Rostral_* = 0.0034; *P_Caudal_* = 0.0120; Figure S4D-E).

Furthermore, on the 21^st^ day post-SCI, rTSMS attenuated motor and sensory dysfunction by BBB scale and heat pain test. However, the neuroprotective effect of rTSMS was suppressed by 2,3 cGAMP (BBB:* P* < 0.001; Latency: *P* < 0.001; Fig. 8B-C). In addition, both the ladder test and the Rivlin test yielded similar outcomes (Fig. 8D-E). Meanwhile, gait analyses showed that rTSMS treatment increased the regularity index while decreased the print position compared to the Ctrl group. However, 2,3 cGAMP reversed these effects (Fig. 8F-G).

In addition, immunofluorescence staining suggested that rTSMS increased SYN expression near the spinal cord lesion after SCI, whereas 2,3 cGAMP treatment downregulated SYN expression. We also observed that microglia in the rTSMS group presented a more ramified morphology, characterized by increased process length and more branches, whereas 2,3 cGAMP treatment reversed these morphological changes induced by rTSMS. More importantly, the immunofluorescence results indicated that microglia phagocytosed many synapses after SCI, and rTSMS markedly alleviated this synapse phagocytosis in microglia. However, compared with those in the rTSMS treatment group, the area and intensity of SYN in microglia were significantly greater after 2,3 cGAMP treatment (Fig. 8H). Collectively, these findings demonstrated that rTSMS alleviates neurological dysfunction and synapse loss in the injured spinal cord by modulating microglial activity through the cGAS-STING signaling pathway.

## Discussion

**Figure 9:**
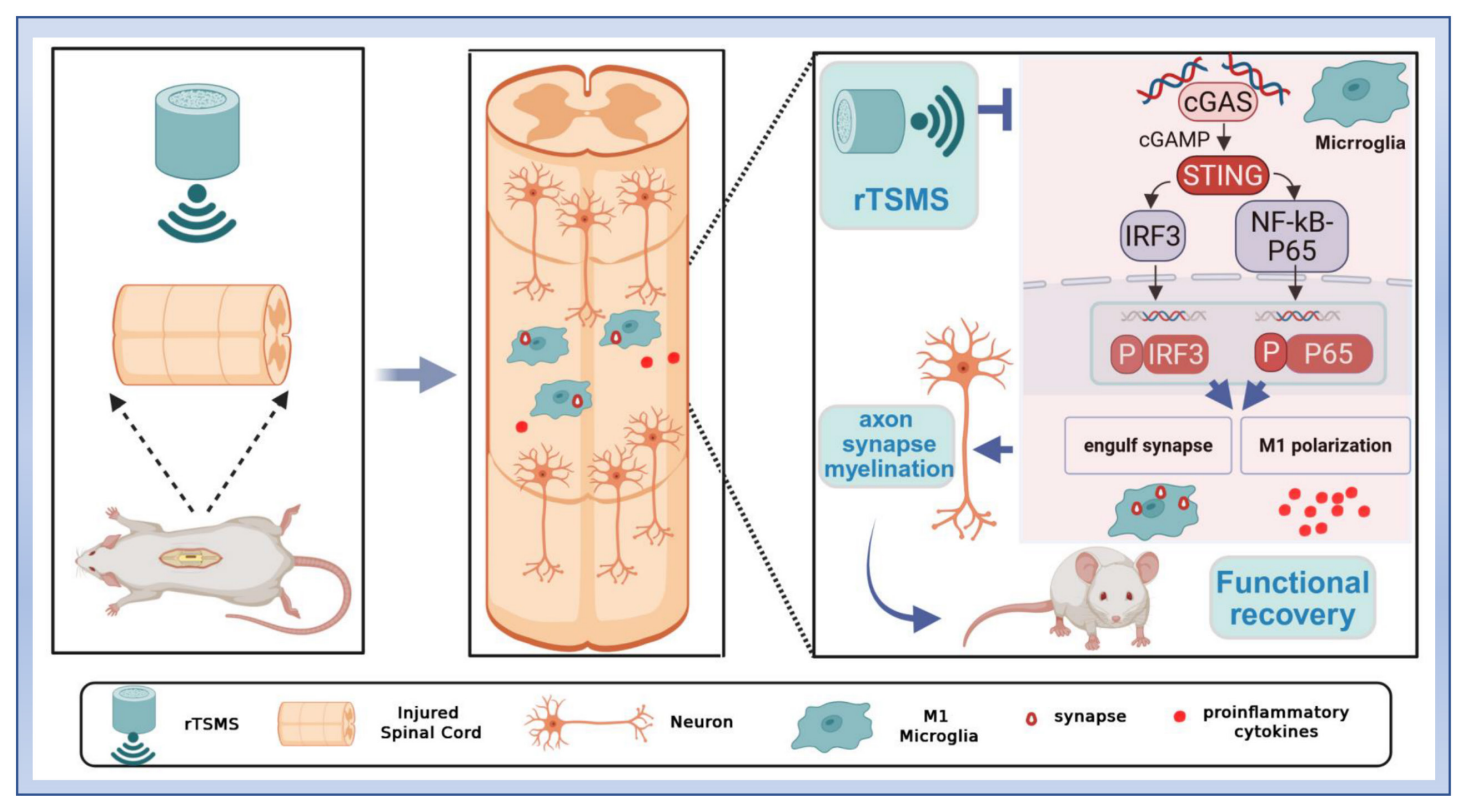
Repetitive trans-spinal magnetic stimulation (rTSMS) inhibits the phagocytosis of synapses by M1 microglia and the release of inflammatory factors through the cGAS-STING signaling pathway, thus improving synapse restoration, axonal regeneration, and functional recovery in a rat model of SCI.

In this study, we found that low-frequency rTSMS, initiated in the sub-acute phase after SCI, leads to more significant improvements in structural and functional recovery compared with high-frequency rTSMS. Low-frequency rTSMS effectively improved the inhibitory environment and facilitated synapse reservation. Importantly, we innovatively revealed that low-frequency rTSMS markedly suppresses microglial phagocytosis of synapses by modulating M1 polarization. Furthermore, we clarified that targeting the cGAS-STING pathway is crucial for inhibiting microglia-mediated synapse loss and the neuroinflammatory response following rTSMS treatment (Fig. 9).

Both low-frequency and high-frequency rTMS exhibit the efficacy for stroke rehabilitation 57, 58. Following stroke, low excitability occurs in the impaired hemisphere, whereas pathological overexcitability is noted in the unaffected hemisphere due to the imbalance of interhemispheric inhibition 59. Theoretically, low-frequency rTMS reduces excitability, whereas high-frequency rTMS increases the excitability of the targeted brain region. Therefore, these methods involving low-frequency rTMS on the uninjured hemisphere and high-frequency on the injured hemisphere are widely used in stroke therapy 60. However, while the model of interhemispheric inhibition is present in the brain, it is absent in the spinal cord. The frequency of rTSMS used to treat SCI may differ from that used to treat stroke. To our knowledge, this is the first study to compare the effects of low-frequency and high-frequency rTSMS, initiated in the sub-acute stage, on SCI.

Our findings showed that, compared with high-frequency rTSMS, low-frequency rTSMS significantly accelerates the recovery of motor and sensory functions, as demonstrated by a series of behavioral tests. Additionally, low-frequency rTSMS protected spinal cord tissue and surviving neurons in the harsh microenvironment induced by SCI. Consistent with our findings, a study reported that low-frequency rTSMS treatment initiated 3 days after injury has a protective effect on neurological function in mice 61. Meanwhile, Ahmed *et al.* suggested that high-frequency rTSMS treatment initiated in the early phase might overexcite the injured spinal cord tissue 22. In high-frequency rTMS treatment, the skull protects the brain from damage caused by overly high-frequency or high-intensity magnetic stimulation. However, in the SCI model, the rat vertebral plate is removed by dorsal laminectomy to expose the spinal cord, making high-frequency magnetic stimulation potentially less optimal for SCI treatment.

Interestingly, Kathe *et al.* reported that spatiotemporal epidural electrical stimulation contributes to the recovery of walking in individuals with SCI and that this mechanism is associated with the inhibition of neuronal activity in the lumbar spinal cord 62. In the current study, electrophysiological data suggested that low-frequency rTSMS results in higher amplitudes of MEPs at 14 and 21 dpi. We speculated that low-frequency rTSMS might decrease the excitation threshold of neurons in the lumbosacral spinal cord. However, more comprehensive electrophysiological evaluation including electroencephalogram, electromyography, sensory evoked potentials, and central motor conduction time are need to study to demonstrate neural circuit restoration following rTSMS treatment.

Neural regeneration after SCI is a complex process, with axonal and synapse formation playing crucial roles in restoring neurological function 30. However, little is known about the role of rTSMS in axonal and synaptic preservation after SCI. In the present study, we clarified the effect of low-frequency rTSMS in modulating axonal regeneration and synapse restoration following SCI. Moreover, Kandhavivorn *et al.* uncovered that low-frequency magnetic treatment promotes axonal regenerative sprouting of cultured spinal motoneurons *in vitro* 63. Our research revealed the excellent performance of low-frequency rTSMS in accelerating myelinated axon regeneration and synapse formation after SCI.

In recent years, growing evidence suggested that SCI leads to chronic microglial activation which contributes to neurodegeneration in various brain regions 64-66. In this study, we also demonstrated that the neuronal activity in cortex is inhibited in the chronic phase following SCI. Interestingly, Ridder *et al.* uncovered that spinal cord stimulation alleviates neuropathic pain by modulating neuronal activity in the primary and secondary somatosensory cortex, pregenual anterior cingulate cortex, and posterior cingulate cortex 67. In order to explore the effects of rTSMS on the brain function, the neuronal activity and the number of Nissl bodies in M1 and S1 regions were evaluated. Our experimental data indicated that rTSMS markedly improves the neuronal activity and increases the number of Nissl bodies in M1 and S1 regions. However, it remains unclear that the effects of rTSMS on the other brain regions such as hypothalamus and thalamus. Further study is needed to explore the mechanisms of brain function reorganization after rTSMS treatment.

After demonstrating the neuroprotective effects of low-frequency rTSMS on SCI, we further investigated the cellular and molecular mechanisms involved. Growing evidence indicates that magnetic stimulation modifies various cell types, including astrocytes, macrophages, microglia, oligodendrocytes, and NPCs, to treat CNS disorders 3, 21, 68-70. In the present study, we found that microglia are the target cells that most actively respond to rTSMS, as revealed by the sc-RNA-seq results. These results indicated that rTSMS treatment significantly reduces the number of microglia in the chronic phase following SCI. Microglia are important cells that produce various immune mediators during both the acute and chronic phases after SCI 71. Our findings revealed that microglia remain overactive at 21 days after SCI, and rTSMS treatment significantly reduces M1 microglial activation.

Microglia-mediated neuroinflammation is known to play a significant role in creating the adverse microenvironment resulting from SCI. Previous studies have focused primarily on the relationship between magnetic stimulation and inflammatory mediators produced by microglia 72, 73. Importantly, in addition to microglia-induced neuroinflammation, microglial phagocytosis is considered another important factor in nerve injury disorders 74. As professional phagocytes, microglia phagocytose axonal or myelin debris in multiple sclerosis and Alzheimer's disease 75. In addition, numerous microglia rapidly migrate to the lesion region to engulf dead cells and myelin debris following SCI 76. The clearance of dead cells and myelin debris creates a favorable microenvironment that enhances nerve regeneration and functional recovery 77. However, a study revealed that overactivated microglia enhance synapse loss in the medial prefrontal cortex 8. These findings suggested that microglial phagocytosis is a double-edged sword. Recent research has shown that high-frequency magnetic stimulation promotes microglial phagocytosis to remove myelin debris 3. In our study, we demonstrated that low-frequency rTSMS suppresses synapse phagocytosis through the modulation of microglial activation. Moreover, Yip *et al.* reported that docosahexaenoic acid confers neuroprotection by preventing microglial phagocytosis via miR-124 78. Thus, microglial phagocytosis is a complex process, and the effects of low- and high-frequency magnetic stimulation on phagocytic activity may differ.

Further studies were conducted to explore the molecular mechanism of microglial phagocytosis following rTSMS treatment. Emerging research suggests that SCI leads to the accumulation of cytoplasmic dsDNA, which activates the cGAS-STING signaling pathway and then induces a neuroinflammatory response 56. Importantly, our sc-RNA-seq analysis identified the cGAS-STING pathway as a potential target for regulating another function of microglia, microglial phagocytosis. Previous studies have indicated that the expression of cGAS and STING increases after SCI, peaking on day 7 (the sub-acute phase) 56. In the current study, we found that the cGAS-STING pathway remains activated in the chronic phase after SCI. More importantly, we demonstrated that magnetic stimulation inhibits M1 microglial overactivation and then mediates microglial phagocytosis of synapses via the cGAS-STING pathway both *in vitro* and in an SCI rat model. Furthermore, another study showed that targeting the cGAS-STING axis promotes microglial polarization to the M2 phenotype and increases the production of anti-inflammatory factors following SCI 79.

In clinical practice, rTSMS has emerged as a minimally invasive neuromodulation strategy for CNS disorders. A randomized, controlled trial demonstrated that 5 Hz rTSMS (twice a week, 4 weeks) alleviates neurological impairment in individuals with Parkinson's disease (PD) 80. Meanwhile, Arii *et al.* assessed the short-term effects of rTSMS on camptocormia, a severe postural abnormality, in PD patients. They observed that 5 Hz rTSMS (40 stimuli total) is effective for improving motor symptoms as well as postural abnormalities 81. Fawaz *et al.* also indicated that 20 Hz rTSMS improves posture, functional ambulation, and the risk of falls in patients with multiple sclerosis 82. However, until now, little is known about the effects of rTSMS on individuals with SCI.

After traumatic SCI, anatomical and pathological changes vary substantially among patients. A recent functional magnetic resonance imaging (fMRI) study showed that the connectivity between the brainstem and spinal cord differs, which is closely related to the level/severity of injuries 83. Furthermore, multimolecular interactions and recovery mechanisms are complex in different phases (acute, sub-acute, and chronic) 84. Chalfouh *et al.* demonstrated that rTSMS increases proliferation and differentiation of spinal cord stem cells during the early phase after SCI 73. However, a recent study indicated that rTSMS modulates lesion scar by decreasing fibrosis in chronic SCI phases 61. Thus, rTSMS stimulation parameters (e.g., frequency, intensity, pulse waveform) and treatment duration may need to be tailored to each patient based on their residual neuronal substrates and functional status to achieve optimal therapeutic effects 85, 86.

Importantly, there are limitations in the present study. For instance, we explored the effects of rTSMS at different frequencies on SCI, other parameters of rTSMS, such as intensity and duration, require further investigation. In addition, we revealed the relationship between low-frequency rTSMS and microglial phagocytosis of synapses. It is vital to explore whether low-frequency rTSMS regulates the phagocytosis of neurons or other cellular components. Furthermore, although sc-RNA-seq analyses and STING agonist intervention data indicated that cGAS-STING pathway might be involved in rTSMS-induced neuroprotection, they are not sufficient to constitute direct mechanistic evidence or a complete logical pathway. Therefore, evaluation of the activity of the key cGAS enzyme and oligomerization/transport of STING is important and necessary. Moreover, the application of genetic techniques serves as the crucial evidence demonstrating that the effect of rTSMS is specifically dependent on the cGAS-STING pathway. Last but not least, according to our pre-clinal research, low-frequency rTSMS might be the promising treatment option for SCI. However, further vigorous studies are needed to determine the therapeutic effect of low-frequency rTSMS on individuals with SCI in the future.

In summary, our study suggests that low-frequency rTSMS, which is initiated in the sub-acute phase after SCI, ameliorates M1 microglial activation and then inhibits microglial phagocytosis of synapses and the neuroinflammatory response via the cGAS-STING pathway. This modulation leads to enhanced neuronal activity, the restoration of synapses and myelinated axons, and ultimately, the promotion of functional recovery. The present research explores new targets for improving SCI prognosis and provides novel insights into the mechanisms by which rTSMS protects against SCI.

## Supplementary Material

Supplementary figures and tables.

## Abbreviations

SCI: spinal cord injury
rTSMS: repetitive trans-spinal magnetic stimulation
cGAS: cyclic GMP-AMP synthase
CNS: central nervous system
TNFα: tumor necrosis factor α
iNOS: inducible nitric oxide synthase
IL-1β: interleukin 1β
Arg1: arginase 1
rTMS: repetitive transcranial magnetic stimulation
SD: Sprague-Dawley
NIH: National Institutes of Health
2,3 cGAMP: 2',3'-Cyclic guanosine monophosphate-adenosine monophosphate sodium
PBS: phosphate-buffered saline
HE: hematoxylin-eosin staining
PFA: paraformaldehyde
BBB: basso, beattie and bresnahan
LBS: ladder beam score
MEPs: motor-evoked potentials
LH: left hindlimb
RH: right hindlimb
NF200: neurofilament-200
SYN: synapsin I
CGRP: calcitonin gene-related peptide
TH: tyrosine hydroxylase
MBP: myelin basic protein
DAPI: 4',6-diamidino-2-phenylindole
RIPA: radioimmunoprecipitation assay
PVDF: polyvinylidene difluoride
PSD95: postsynaptic density protein 95
P65: NF-kB-P65
p-P65: p-NF-kB-P65
TBST: tris-buffered brine tween20
sc-RNA-seq: single-cell RNA sequencing
UMI: unique molecular identifier
PCA: principal component analysis
DEGs: differentially expressed genes
GO: Gene Ontology
KEGG: Kyoto Encyclopedia of Genes and Genomes
GSEA: Gene Set Enrichment Analysis
qRT-PCR: quantitative real-time PCR
P2: postnatal day 2
PDL: poly-D-lysine
MEM: minimum essential medium
FBS: fetal bovine serum
MS: magnetic stimulation
LPS: lipopolysaccharide
ANOVA: analysis of variance
DPI: days post-injury
RF: right forelimb
RH: right hindlimb
NPCs: neural progenitor cells
PD: Parkinson's disease
fMRI: functional magnetic resonance imaging
