# Supplementary material for: Repetitive Trans-spinal Magnetic Stimulation Suppresses Microglia to Engulf Synapse and Promotes Nerve Repairment via cGAS-STING Signaling Pathway after Spinal Cord Injury
Source: Int J Biol Sci. 2025 Sep 29;21(14):6132–55. doi: 10.7150/ijbs.114428 (PMC12594571; doi:10.7150/ijbs.114428)
Supplement: Supplementary file 1 — Supplementary figures and tables. [file ijbsv21p6132s1.pdf]

Supplementary Figures

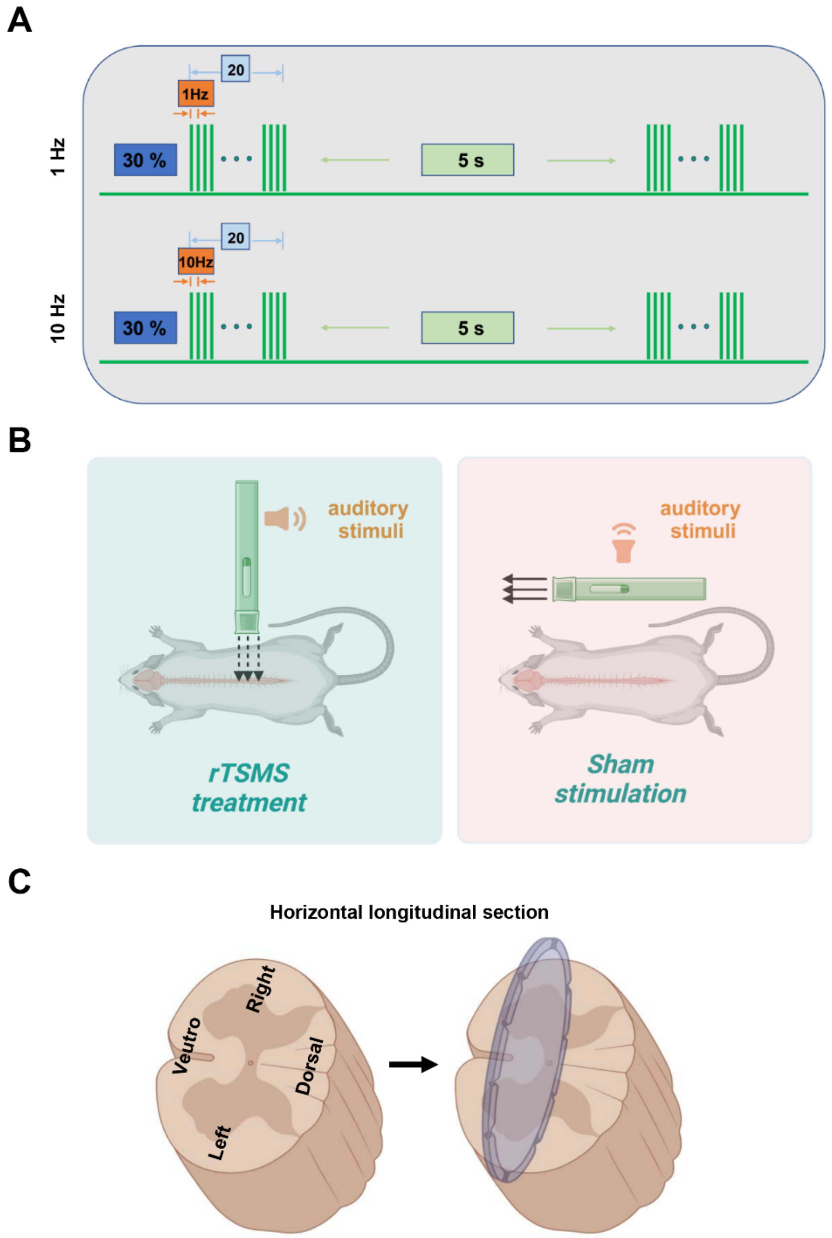

Figure S1. Low-frequency rTSMS ameliorates lesion area and promotes neuronal survival in rats with SCI. (A) The 1Hz low-frequency rTSMS and 10Hz high-frequency rTSMS parameters. (B) The position of coil during rTSMS treatment (left) and sham stimulation (right). Image was created with BioRender.com. (C) Schematic illustration of horizontal longitudinal section of the spinal cord. Image was created with BioRender.com.

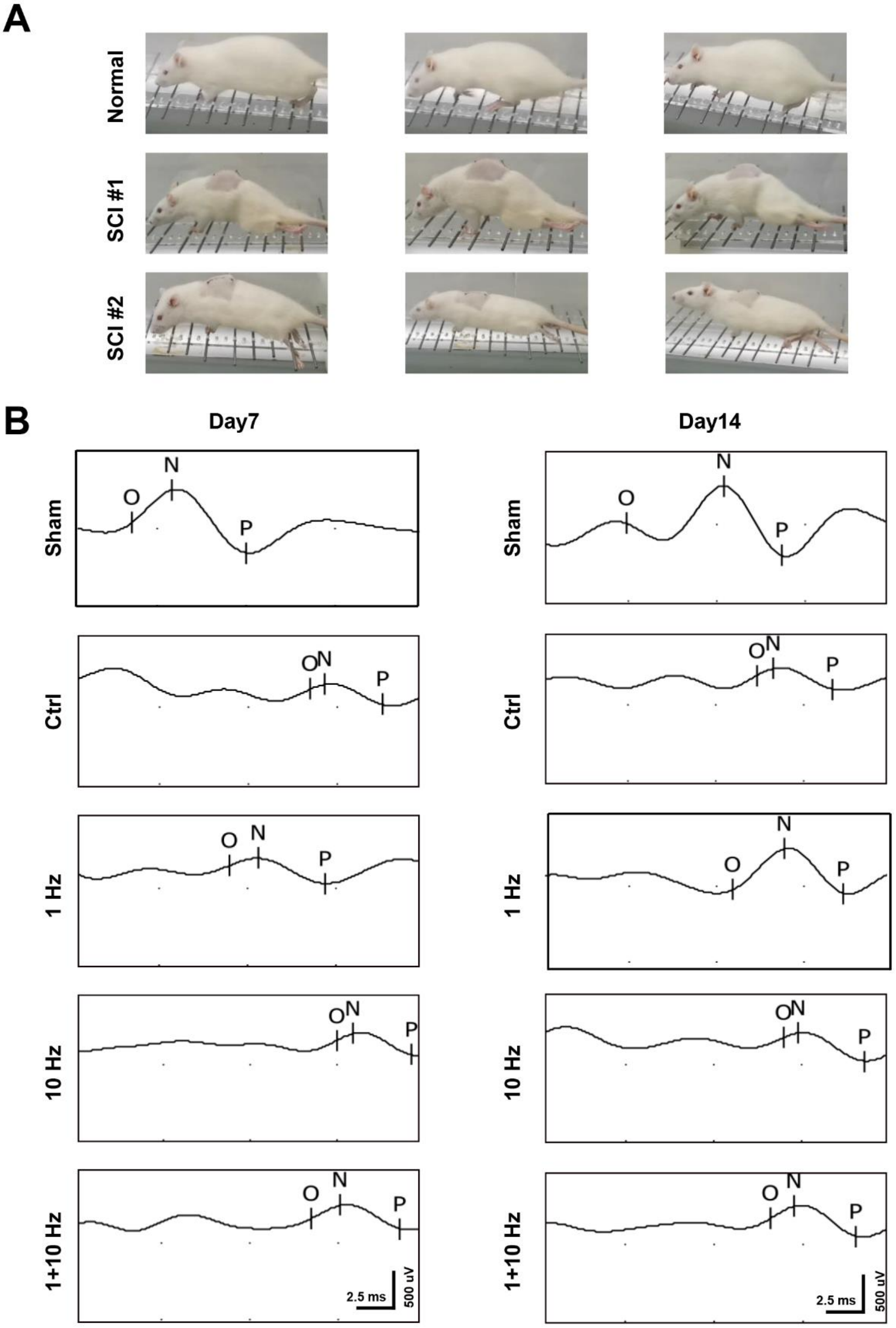

Figure S2. Low-frequency rTSMS enhances motor and sensory function recovery following SCI. (A) Rats in the horizontal ladder test before and after SCI operation. (B) Representative individual electrophysiological traces at 7 and 14 dpi.

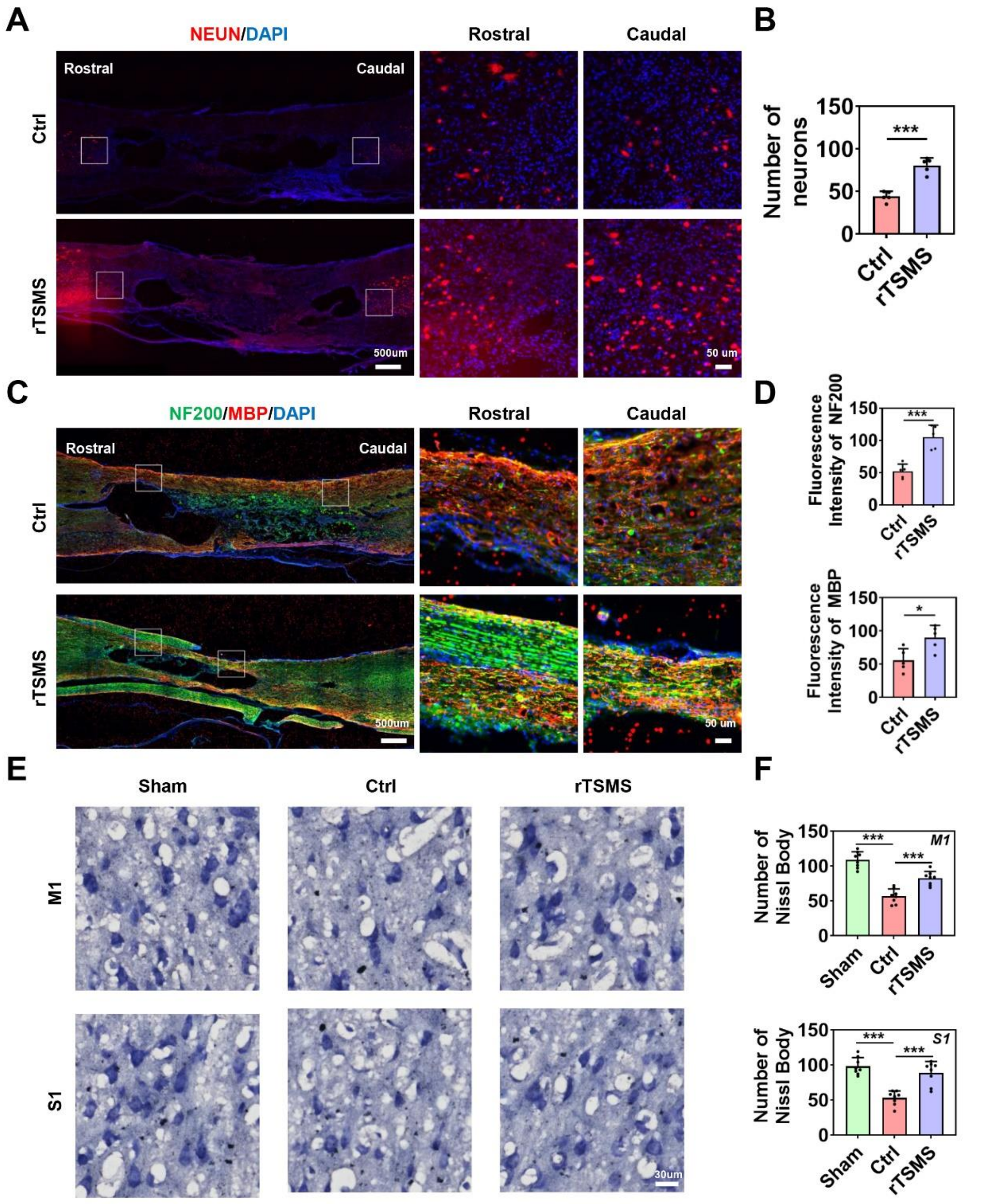

Figure S3. rTSMS enhances synapse formation and neuronal activity in rats with SCI. (A-B) Representative images of immunostaining and statistical analysis of NEUN (red) and DAPI (blue) in the injured spinal cord at 21 dpi, n = 5. (C-D) Representative images of immunostaining and statistical analysis of NF200 (green), MBP (red) and DAPI (blue) in the injured spinal cord at 21 dpi, n = 5. (E-F) Representative images and statistical analysis of Nissl body in the M1 and S1 regions at 21 dpi. n = 8. *$P < 0.05$, ***$P < 0.001$.

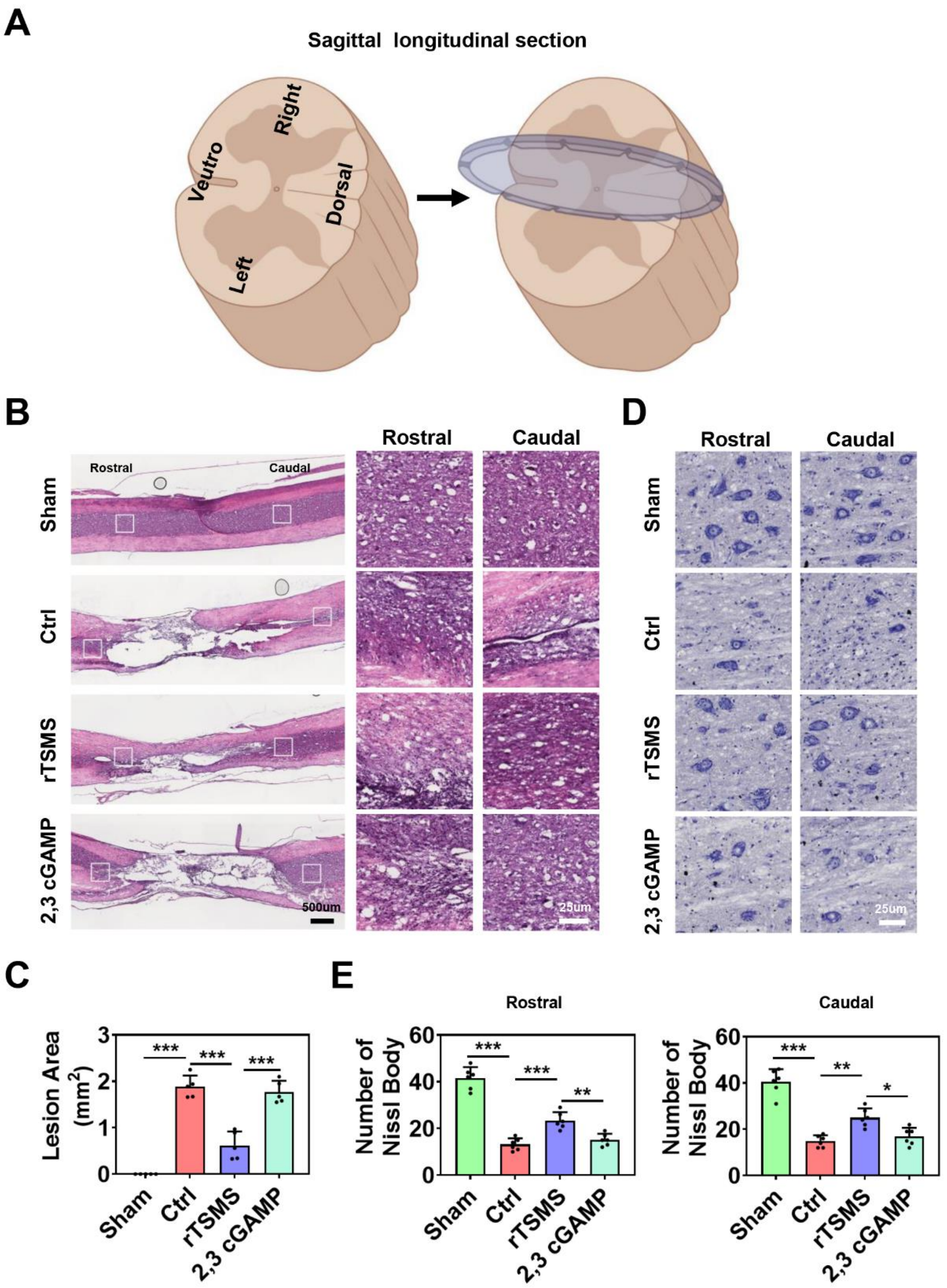

Figure S4. STING agonist diminishes the neuroprotective effects of rTSMS in SCI rats. (A) Schematic illustration of sagittal longitudinal section of the spinal cord. The image was created with BioRender.com. (B-C) Representative HE-stained images and quantification of the lesion area in rats at 21 dpi, n = 5. (D-E) Representative Nissl-stained images and quantification of Nissl body at 21 dpi, n = 6. $^{*}P < 0.05$, $^{**}P < 0.01$, $^{***}P < 0.001$.
